# Aberrant Splicing Signatures Underpin Oligodendrocyte Damage in ALS and Neuron Loss in FTD

**DOI:** 10.1002/advs.202514886

**Published:** 2026-02-04

**Authors:** Chen Du, Yinming Li, Rong Wu, Yufei Shen, Jiayi Yang, Xuan Xiao, Yu Zhou

**Affiliations:** ^1^ TaiKang Center For Life and Medical Sciences Hubei Provincial Research Center for Basic Biological Sciences Hubei Key Laboratory of Cell Homeostasis RNA Institute College of Life Sciences Wuhan University Wuhan China; ^2^ Renmin Hospital Wuhan University Wuhan China; ^3^ Department of Physiology Pharmacology and Therapeutics Brain Science Institute Johns Hopkins University School of Medicine Baltimore Maryland USA; ^4^ Frontier Science Center for Immunology and Metabolism State Key Laboratory of Virology Wuhan University Wuhan China

**Keywords:** aberrant splicing, ALS, biomarker, FTD

## Abstract

Amyotrophic lateral sclerosis (ALS) and frontotemporal dementia (FTD) are two severe diseases sharing similar genetic, pathological, and clinical features, including TDP‐43 pathology. However, differences in molecular changes between ALS and FTD remain elusive. Here, integrating large sets of bulk and single‐nucleus RNA‐seq from ALS/FTD patients revealed expression and splicing changes indicating more severe oligodendrocyte damage in ALS than FTD, and more significant neuron loss in FTD. Specifically, we identified 31 oligodendrocyte‐specific and 507 neuron‐specific aberrant splicing junctions as potential biomarkers with robust classification performance, and experimentally validated a novel target in patient tissues. Moreover, we found that abnormally spliced transcripts produced de novo peptides in patients’ cerebrospinal fluids. Importantly, we further identified the targets of TDP‐43 in glial cells and decoded the differential RNA‐binding protein (RBP) contexts of TDP‐43‐regulated aberrant splicing. These findings uncover that ALS and FTD patients have distinct dysfunctional cell populations harboring specific aberrant splicing signatures, suggesting varying cellular impacts and providing potential biomarkers and insights into molecular mechanisms underlying ALS/FTD.

## Graphical abstract

Integrating bulk and single‐nucleus RNA‐seq from ALS and FTD patients, we revealed distinct cellular dysfunctions, with severe oligodendrocyte damage in ALS and pronounced neuron loss in FTD. We identified cell‐type‐specific aberrant splicing events as potential biomarkers with robust classification performance, some of which produce de novo peptides in cerebrospinal fluid, and decoded the differential RNA‐binding protein contexts underlying ALS/FTD.

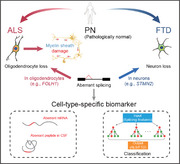

## 1 Introduction

Amyotrophic lateral sclerosis (ALS) is a neurodegenerative disease characterized by the degeneration of upper and lower motor neurons. In contrast, frontotemporal dementia (FTD) represents a prevalent form of dementia associated with progressive neuronal degeneration, particularly in the frontotemporal neural networks. Clinically, cognitive and behavioral abnormalities are observed in 50% of ALS patients, with approximately 13% presenting concomitant FTD [1]. Motor neuron damage is found in up to 40% of individuals diagnosed with FTD, whereas ALS occurs in 12.5% of FTD cases [2, 3, 4]. Furthermore, both diseases share common pathogenic genes, such as C9ORF72, and TDP‐43 pathological features present in 97% of ALS patients and 45% of FTD [5, 6]. Based on this clinical and genetic similarity, ALS and FTD are currently regarded as distinct phenotypes within the same spectrum of neurodegenerative diseases; however, their underlying mechanisms remain unclear [7].

Unraveling the molecular differences between ALS and FTD contributes to a better understanding of the underlying pathogenic mechanisms and assists in disease diagnosis and treatment. Numerous high‐throughput sequencing data provided clues to explain the pathogenesis of ALS/FTD; many studies have previously dissected the pathogenic mechanisms based on omics data, including RNA‐seq [8, 9], snRNA‐seq [10, 11, 12, 13, 14, 15], and genome sequencing [16]. While these studies provided valuable insights, most of them did not distinguish ALS and FTD or only focused on one of the two, despite having patient data with clear clinical diagnoses. A recent work by Pineda et al. globally compared changes between ALS and FTD based on ALS/FTD patients’ snRNA‐seq data and identified vulnerable cell types and disease mechanisms across different cell types, recognizing that cell‐type‐specific alterations across ALS and FTD in the same brain region are conserved [11]. However, the non‐conserved changes between the two diseases were neglected, and current knowledge of molecular distinctions between ALS and FTD is still incomplete.

To bridge this gap, we integrated patients’ bulk RNA‐seq and snRNA‐seq data to investigate the potential molecular and cellular mechanism differences between ALS and FTD. Specifically, we elucidated the gene expression alterations observed in bulk RNA‐seq by considering changes in cell composition. Ultimately, we found a correlation between TDP‐43 pathology‐mediated disease‐specific aberrant splicing junctions and cell loss. We observed that oligodendrocytes are impacted more severely in the cortex of ALS patients; in contrast, neurons are more severely affected in FTD patients, along with corresponding cell loss. Additionally, we uncovered that other RBPs were involved and facilitated the cell‐type‐specific aberrant splicing events. The analysis of the aberrant junctions highlighted that the frontotemporal cortex is affected in both FTD and ALS. We further identified cell‐type‐specific aberrant splicing signatures that could distinguish ALS and FTD patients well. Notably, several abnormal spliced transcripts were found to generate de novo proteins, which could be detected by mass spectrometry (MS) in the cerebrospinal fluid (CSF) of ALS/FTD patients.

The novelty of our research lies in the new insights drawn from previously reported findings, offering a fresh perspective compared to existing studies. For instance, while Humphrey et al. focused on oligodendrocyte damage in the spinal cords of ALS patients [17], our study expanded the scope to the cortex and highlighted distinctions between ALS and FTD. Furthermore, we identified cell‐type‐ and disease‐specific aberrant splicing junctions as biomarkers for ALS and FTD, which may promote early diagnosis and guide targeted therapies. Additionally, regarding CSF peptides derived from TDP‐43 pathology, Irwin et al. concentrated on detecting a single target (*HDGFL2*) [18] and Seddighi et al. focused on screening induced neurons in vitro [19], but our work systematically screened clinical sample data, potentially offering higher clinical translational value.

In sum, our results reveal molecular differences between ALS and FTD, highlight the involvement of oligodendrocytes in ALS and neurons in FTD, and identify potential biomarkers, with an oligodendrocyte‐specific aberrant splicing junction validated in patient tissues.

## 2 Results

### 2.1 Gene Expression Trends in the Cortex of ALS and FTD Patients Are Opposite

**FIGURE 1:**
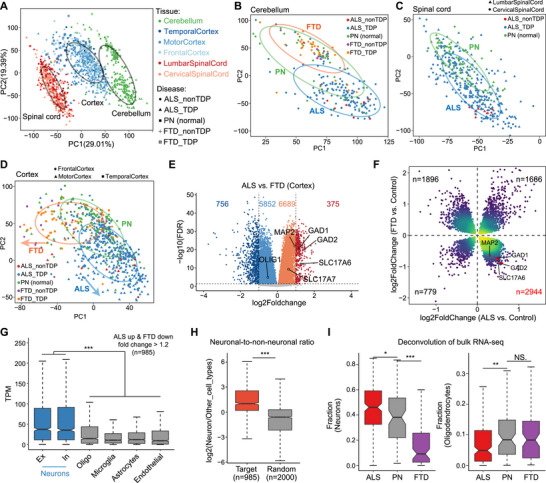
Expression atlas of ALS/FTD patients in multiple tissues. (A) PCA analysis of the expression patterns of all ALS/FTD patients and control samples. The colors of dots represent tissues, and the shapes of dots indicate disease types. Three black ovals indicate 60% confidence intervals of cerebellum, cortex, and spinal cord samples, respectively. (B‐D) PCA plots showing expression patterns in the cerebellum (B), spinal cord (C), and cortex (D) samples respectively. The colors of the dots represent disease types and the shapes of the dots indicate sub‐tissue types. Green ovals indicate 60% confidence intervals of control samples, blue ovals indicate 60% confidence intervals of ALS samples, and orange ovals indicate 60% confidence intervals of FTD samples. (E) Volcano plot showing differential gene expression between ALS and FTD in the frontal and temporal cortex. (F) Scatter plot showing fold change of DEGs in the frontal and temporal cortex of ALS and FTD. (G) Box plot showing cell‐type‐specific gene expression pattern of cortical genes up‐regulated in ALS but down‐regulated in FTD. (H) Box plot comparing the neuronal‐to‐non‐neuronal expression ratios of cortical genes up‐regulated in ALS but down‐regulated in FTD, compared to random gene sets. (I) Box plots showing the fraction of neurons (left) and oligodendrocytes (right) obtained through deconvolution in cortex samples from various patients.

To study the expression pattern of ALS and FTD patients, we leveraged a public ALS and FTD patients’ bulk RNA‐seq dataset from the New York Genome Center (NYGC) ALS Consortium (GSE153960) of multiple tissues, including cerebellum, cortex, and spinal cord, in which patients were grouped according to whether TDP‐43 inclusion existed (Figure S1A). First, we performed principal component analysis (PCA) on the above samples (*n* = 1603) based on the expression of the top 10,000 genes with the largest standard deviation (SD) to explore global expression relationships. The result shows that the samples are mainly clustered into three large clusters by tissues (cerebellum, spinal cord, and cortex) rather than by disease types (Figure 1A).

Notably, samples are largely clustered together by disease types within specific tissues (Figure 1B–D). In the cerebellum, ALS patients show more obvious differences from pathologically normal (PN) controls than FTD patients (Figure 1B). This phenomenon is consistent with the fact that ALS affects motor function more severely than FTD due to the cerebellum's role in motor behavior control [20], and cerebellar degeneration has been confirmed in ALS patients [21]. In the spinal cord group, there are only samples of ALS patients, and it could be seen that there are certain differences between ALS patients and controls (Figure 1C), and differentially expressed genes (DEGs) were mainly up‐regulated in the spinal cord which related to immune response (Figure S1B,C), possibly due to previously reported microglial activation in ALS spinal cord and lower motor neuron degeneration [17].

Most interestingly, we found that gene expression changes in the cortex appear to occur in two different directions in ALS and FTD patients relative to controls in PCA analysis (Figure 1D). To directly compare the differential gene expression profiles between ALS and FTD in the cortex, we performed a head‐to‐head comparison of their transcriptomic data (Figure 1E), using only frontal and temporal cortex samples available for both ALS and FTD to ensure the reliability of the analysis. This revealed more pronounced gene expression differences than those observed when each disease was compared individually to the control (Figure S1D,E). Furthermore, ALS and FTD exhibited opposite changes relative to the normal control of the same gene in the cortex. The differences between the two diseases, therefore, appeared to result from the cumulative effect of these opposing alterations (Figure S1F). This further supports the hypothesis that ALS and FTD represent two extremes along a divergent spectrum of disease progression, with distinct and opposing patterns of cortical gene expression relative to controls.

### 2.2 Cellular Composition Shifts Drive Cortical Expression Differences in ALS and FTD

In addition, we examined the distribution of DEGs with FDR < 0.05 in the cortex of ALS and FTD patients relative to controls (Table S1). The majority of these genes were up‐regulated in ALS but down‐regulated in FTD, a total of 2,944 genes, including several neuron‐specific marker genes, such as *MAP2* and *SLC17A6* (Figure 1F), consistent with those up‐regulated in ALS compared to FTD (Figure 1E). Upon applying a fold‐change threshold of 1.2, we again identified the largest set of overlapping cortical DEGs as those up‐regulated in ALS and down‐regulated in FTD, comprising 985 genes in total (Figure S1G). The 985 genes were significantly enriched in pathways related to multiple neurodegenerative diseases, such as Parkinson's disease, and the function of synapses (Figure S1H), suggesting their critical role in the progression of neurodegenerative diseases. To explore expression features of the 985 genes, we utilized a single‐cell‐based cell type gene expression matrix collected in The Human Protein Atlas (https://www.proteinatlas.org), which provides normalized expression levels for individual genes across various cell types, including excitatory neurons (Ex), inhibitory neurons (In), astrocytes, oligodendrocytes, microglia, endothelial cells, and others. By extracting the expression values of the 985 genes across these cell types, we found that these genes are highly expressed in neurons, including Ex and In (Figure 1G). To ensure that this observation was not simply due to generally higher gene expression in neurons, we calculated the expression ratio of each gene between neurons and all other cell types. Compared to a randomly selected set of 2000 genes, the 985 genes up‐regulated in ALS and down‐regulated in FTD showed significantly higher neuron‐to‐non‐neuronal expression ratios (Figure 1H). This indicated a specific enrichment of these genes in neurons, rather than a non‐specific effect of globally higher neuronal expression levels.

We hypothesized that the observed changes in their expression levels at the bulk RNA‐seq level were driven by alterations in cellular composition rather than transcriptional repression. To further investigate this, we performed deconvolution analysis of bulk RNA‐seq data using DWLS [22] based on cell‐type‐specific expression profiles of neurons and glial cells obtained from the Human Protein Atlas. The results revealed a significant reduction in the proportion of neurons in FTD, whereas oligodendrocyte abundance was markedly decreased in ALS (Figure 1I). This finding was consistent with our observation that the oligodendrocyte‐specific marker gene *OLIG1* was significantly down‐regulated in ALS (Figure 1E).

Taken together, these findings support the hypothesis that cortical gene expression changes are closely linked to neurodegenerative disease progression and follow opposing patterns in ALS and FTD. These differences are largely attributable to shifts in cortical cellular composition, with a reduction in neuronal populations in FTD and a loss of oligodendrocytes in ALS.

### 2.3 snRNA‐seq Reveals Changes in Cellular Composition in ALS/FTD Patients’ Cortex

**FIGURE 2:**
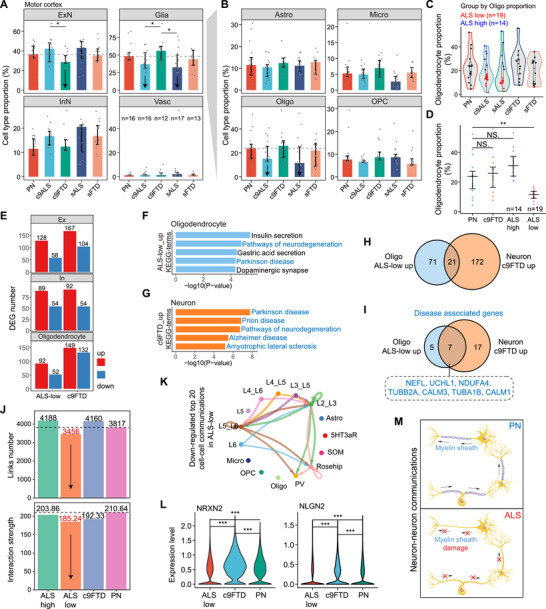
Cell proportion, gene expression, and cell‐cell communication analysis of ALS/FTD patients’ cortex snRNA‐seq. (A) Bar plots showing the median proportion of 4 main cell groups in PN, ALS, and FTD patients’ motor cortex. Error bars representing the 25^th^ and 75^th^ percentiles. (B) Bar plots showing the median proportion of 4 subclusters of glial cells in PN, ALS, and FTD patients’ motor cortex. Error bars representing the 25^th^ and 75^th^ percentiles. (C) Violin plot showing the distribution of oligodendrocyte proportions across motor cortex snRNA‐seq samples in sporadic (s) and C9orf72 (c9) ALS patients, sporadic (s) and C9orf72 (c9) FTD patients, and the normal controls (PN). Each sample's value is marked with an individual point. (D) Scatter plot showing the proportion of oligodendrocytes in PN, ALS‐low, ALS‐high, and c9FTD motor cortex samples. (E) Bar plots showing the number of DEGs between ALS‐low/c9FTD patients and PN, identified in excitatory (Ex), inhibitory (In) neurons, and oligodendrocytes through motor cortex snRNA‐seq. (F‐G) Bar plots showing the top 5 enriched KEGG pathway terms of up‐regulated genes in oligodendrocytes of ALS‐low (F) and up‐regulated genes in neurons of c9FTD (G), respectively. Neurodegenerative disease‐related pathways are marked in blue. (H) Venn plot depicting the intersection of up‐regulated genes identified in oligodendrocytes of ALS‐low and neurons of c9FTD patients. (I) Intersection of neurodegenerative disease‐associated genes identified in oligodendrocytes of ALS‐low and neurons of c9FTD patients. (J) Bar plots showing the number (top panel) and weight (bottom panel) of all links inferred among each group. (K) Circle plot showing the top 20 down‐regulated intercellular communications in ALS‐low compared to PN. The width of each link indicates the absolute difference. (L) Single‐cell gene expression violin plots of NRXN2 and NLGN2. (M) Diagram illustrating the reduction in oligodendrocytes disrupting neuron‐neuron communications in ALS patients.

Since we observed opposite gene expression trends in ALS/FTD patients’ cortex in the above bulk RNA‐seq data, we further utilized a publicly available single‐nucleus RNA sequencing (snRNA‐seq) dataset (PRJNA1073234) of patients’ cortex to directly quantify cell composition in different tissues, including motor cortex and prefrontal cortex. First, we divided cells into four major categories: excitatory neurons, inhibitory neurons, glial cells, and vascular cells, and then counted cell numbers, followed by calculating the cell composition of each disease type. Since neurons in the adult mammalian brain can hardly regenerate [23], we mainly focused on the clusters with a significantly reduced cell proportion, while the clusters with relatively increased cell proportions are probably due to the decrease of other clusters. Patients in this dataset were divided into C9ORF72 mutations (c9ALS/c9FTD) and sporadic ones (sALS/sFTD). To minimize the influence of outliers, we calculated the median proportion of each cell type within each patient group. This analysis revealed a sharp reduction in glial cells in the motor cortex of both c9ALS and sALS patients. In contrast, c9FTD patients exhibited a significant decrease in excitatory neurons (ExN), while sFTD patients showed relatively stable cell‐type composition. Notably, the difference in cellular composition between ALS and FTD is greater than that between either disease and the control group (Figure 2A). The proportions of excitatory neurons in ALS and FTD patients are both reduced in the prefrontal cortex, but the reduction degree in ALS is smaller than that in FTD patients, and moreover, the proportion of glial cells in ALS is smaller than in FTD, suggesting that the loss of glial cells is more serious in ALS (Figure S2A).

To further investigate which glial cell type changed most, especially in the motor cortex, we subdivided glial cells into 4 subclusters: astrocyte, microglia, oligodendrocyte, and oligodendrocyte precursor cell (OPC). At the mean level, oligodendrocytes appeared reduced in both sALS and c9ALS patients (Figure 2B). However, these differences did not reach statistical significance when comparing all ALS patients to controls. Considering the high inter‐patient heterogeneity, and to better understand this discrepancy, we examined the distribution of oligodendrocyte proportions across ALS samples and found a bimodal pattern. Notably, ALS samples with lower proportions of oligodendrocytes showed a more compact distribution pattern, whereas those with higher proportions were more dispersed (Figure 2C, Figure S2B), indicating that cases with reduced oligodendrocyte abundance may represent a biologically more consistent subtype. To better capture the effects of oligodendrocyte loss, we selected the 60% of ALS patients with the lowest oligodendrocyte proportions (*n* = 19), hereafter referred to as the “ALS‐low” group, for downstream analyses. Notably, oligodendrocyte proportions in the ALS‐low group were significantly reduced compared to controls (Figure 2D). In addition, we found that excitatory neurons in cortical layer 5 are mainly reduced in c9 FTD patients (Figure S2C), consistent with previously reported vulnerable cell populations [11].

Then, we calculated the gene expression changes in neurons and oligodendrocytes based on the snRNA‐seq data and found that the altered genes in neurons and oligodendrocytes are mainly up‐regulated in both ALS and FTD (Figure 2E). KEGG enrichment analysis was performed on genes up‐regulated in oligodendrocytes of ALS patients and those up‐regulated in neurons of FTD patients. We found that the enriched terms all related to a variety of neurodegenerative diseases (Figure 2F,G), which all point to cell death at the end of the corresponding KEGG pathways. Although enriched pathways are roughly the same, only 21 genes overlap between the two sets, most of which are independent (Figure 2H). Moreover, seven of the overlapping genes are disease‐associated genes (Figure 2I), among which *UCHL1* has been proposed as a therapeutic target for upper motor neurons in neurodegenerative diseases [24]. We speculated that the up‐regulation of these genes indicates the activation of neurodegenerative disease pathways, thus leading to the death of oligodendrocytes in ALS and neurons in FTD.

Furthermore, we compared the overlap of DEGs identified at the bulk and single‐cell levels in both ALS and FTD. The number of overlapping genes was found to be very limited (Figure S2F,G), further supporting the hypothesis that gene expression changes observed at the bulk RNA level were largely driven by alterations in cellular composition rather than intrinsic transcriptional regulation within individual cells.

In addition to the changes in gene expression, we used NeuronChat [25] to quantitatively analyze changes in cell–cell communication under disease conditions. We randomly selected 20 000 cells from each disease group sample to ensure equal cell counts and prevent bias in statistical analysis due to variations in cell quantity. We found that the number of links and the strength of cell–cell interactions are both significantly reduced in ALS patients with low oligodendrocyte proportion but not in ALS‐high (Figure 2J), and communication weight between various cell types is reduced in the ALS‐low group, especially between neurons (Figure 2K; Figure S2H), further emphasizing the critical role that oligodendrocytes play in cell–cell communications. Additionally, the number of links of most interaction pairs was significantly reduced in ALS‐low patients, particularly those mediated by neurexin family proteins (Figure S2I), which are essential for synapses assembled [26]. We identified significantly down‐regulated ligand–target pairs, such as NRXN2‐NLGN2 and NRXN2‐NLGN3. The expression levels of the ligand and target genes in these pairs were significantly lower in excitatory neurons of ALS‐low patients compared to PN controls (Figure 2L). Interestingly, we detected alterations in the neurexin family in ALS but not in FTD, an unexpected finding given their well‐established role in learning and memory [27]. This discrepancy may reflect synaptic disturbances secondary to motor neuron degeneration in ALS, regional or cohort‐specific differences in FTD, or the possibility that FTD‐related synaptic dysfunction involves alternative molecular pathways. This intriguing difference warrants further investigation to understand potentially distinct mechanisms of synaptic dysfunction in ALS and FTD.

Based on previous studies on the neural circuit degeneration in ALS/FTD patients [28], and oligodendrocytes assembled myelin wrapped around axons in the central nervous system (CNS) in accelerating nerve conduction [29], we propose a model in which oligodendrocytes are substantially lost in the cortex of ALS patients, resulting in myelin sheath damage, followed by disrupted neuron–neuron communications and nerve conduction (Figure 2M), explaining the possible different pathogeneses between ALS and FTD.

### 2.4 Aberrant Splicing Junctions Underpin Different Cell Type Damage in the Cortex of ALS and FTD Patients

TDP‐43 pathology was the hallmark of ALS and FTD [30], and the role of TDP‐43 in splicing regulation has been widely reported. Furthermore, as cryptic exons generated by TDP‐43 depletion were found to contribute to cell death [31], we wondered whether the differences in cell type proportion changes observed in snRNA‐seq data between ALS and FTD are related to aberrant splicing. Thus, we identified significantly changed splicing junctions in patients compared with controls in different tissues using MAJIQ v2 [32] with stringent criteria. For up‐regulated splicing junctions in patients, we defined normal and aberrant junctions based on their usage by PSI in control samples of the same tissue type: PSI < 0.05 as the aberrant type, PSI ≥ 0.05 as the normal type (Figure S3D). We used matched PN samples as a reference rather than genome annotations, as many unannotated junctions are commonly used in normal brain tissue. We demarcated aberrant junctions to be strictly absent in controls but significantly up‐regulated in patients. To further investigate the features of identified aberrant splicing junctions, we first calculated the consensus sequences for the 5′ and 3′ splice sites of these abnormal junctions. We found that the aberrant junctions utilize the classic splice site sequences (GT/AG) in ALS and FTD (Figure S3E), although both exhibit lower splicing strength compared to annotated ones calculated by SpliceAI [33] (Figure S3F). Interestingly, ALS aberrant junctions have even lower strength than those in FTD, particularly at the 3′ splice site (Figure S3F).

**FIGURE 3:**
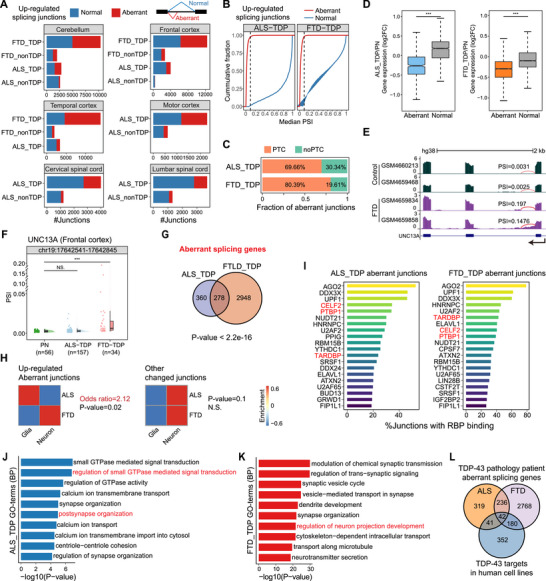
Splicing alterations detected in ALS and FTD through bulk RNA‐seq analysis. (A) Bar plots showing the number of up‐regulated junctions identified in different tissues between patients and PN. Aberrant junctions were shown in red, and normal junctions (normally used in PN) were shown in blue. (B) Cumulative curves of PSI for aberrant junctions and normal junctions in the frontal cortex. Aberrant junctions are shown in red, and normal junctions are shown in blue. (C) Fraction of aberrant junctions predicted to produce transcripts containing a premature termination codon (PTC) or not. (D) Box plots showing expression change of genes containing aberrant junctions and normal junctions in the frontal cortex of ALS‐TDP (left panel) and FTD‐TDP (right panel). The *p*‐values were calculated by the Wilcoxon test. (E) UCSC genome browser view of the aberrant splicing in UNC13A. The quantified PSI values of the aberrant splicing junction are labeled for each sample, respectively. (F) Scatter and violin plots illustrating PSI changes of the aberrant junction in UNC13A among different sample groups in the frontal cortex. The *p*‐values were calculated by the Wilcoxon test. (G) Venn plot depicting the intersection of genes containing aberrant junctions of ALS‐TDP and FTD‐TDP patients in the frontal cortex. The *p*‐value was calculated by Fisher's exact test. (H) Heatmap showing the enrichment of ALS/FTD aberrant junctions and other changed junctions in neurons and glial cells of the frontal cortex. Odds ratios and *p*‐values are calculated by Fisher's exact test. (I) Percentage of aberrant junctions bound by RBPs in ±150 nt region flanking the splice sites in the frontal cortex of ALS‐TDP (left panel) and FTD‐TDP (right panel). (J‐K) Bar plots showing the top 10 enriched BP terms of genes containing aberrant junctions in the frontal cortex of ALS‐TDP (J) and FTD‐TDP (K), respectively. (L) Venn plot depicting the intersection of genes containing aberrant junctions in the frontal cortex of ALS‐TDP, FTD‐TDP, and genes containing up‐regulated novel junctions detected in human cell lines after TDP‐43 knockdown.

Not surprisingly, for each tissue, patients in the TDP group (patients with TDP‐43 inclusion) have more aberrant junction changes than those in the non‐TDP group (patients without TDP‐43 inclusion) in both ALS and FTD (Figure 3A). In addition, we found that the number of changed junctions in FTD patients is much more than that in ALS (Figure 3A; Figure S3G), indicating FTD patients have greater splicing changes than ALS. Since it had been reported that TDP‐43‐mediated cryptic exons would often promote nonsense‐mediated RNA decay (NMD) of the associated transcripts [34], this phenomenon would cause aberrant junctions to be hardly detected in global RNA‐seq. We calculated the PSI value of aberrant and normal junctions and found that PSI values of aberrant junctions were mostly less than 0.1, while the PSI value distribution of normal junctions was relatively even (Figure 3B). Further analysis of aberrant junctions revealed that 70%–80% of them would lead to premature termination codons (PTCs) in corresponding transcripts (Figure 3C), suggesting that those transcripts might undergo NMD and be targeted for degradation [35]. Consistently, these aberrant junctions caused down‐regulated RNA levels of corresponding genes in patients compared to genes containing normal junctions (Figure 3D), functioning in a poisonous manner. For instance, in the previously reported gene *UNC13A* [36, 37], aberrant junctions would lead to the production of transcripts containing a cryptic exon, especially in FTD patients, illustrated by patient examples (Figure 3E) and statistics (Figure 3F). Genes containing aberrant splicing junctions showed considerable overlap between ALS‐TDP and FTD‐TDP, and nearly half of the abnormal genes in ALS‐TDP also exhibited novel junctions in FTD‐TDP (Figure 3G), which may be directly regulated by TDP‐43. These results underscore those aberrant splicing junctions in post‐transcriptional gene regulation.

To figure out which cell types these aberrant junctions occurred in, we first identified specifically expressed genes of various cell types in PN cortex samples through the above snRNA‐seq data and performed Fisher's exact test to assess the enrichment. Surprisingly, in the frontal cortex, aberrant junctions in ALS patients are significantly more enriched in glial cell‐specific genes, while in FTD patients, they are more enriched in neuronal genes (odds ratio > 2), consistent with the observed patterns of cell loss (Figure 3H). We found the same phenomenon in the temporal cortex (Figure S3H). However, other changed junctions do not exhibit this characteristic, showing no significant difference in enrichment patterns between neurons and glial cells in ALS and FTD (Figure 3H). Together, these results suggest that aberrant splicing events in ALS occur in glial cells and are associated with glial loss, particularly of oligodendrocytes, whereas in FTD, they occur in neurons and are in line with neuron loss.

Furthermore, to determine whether observed aberrant splicing was caused by TDP‐43 pathology, we integrated human RNA‐binding proteins (RBPs) CLIP‐seq data collected by the POSTAR3 database [38] and found that a considerable fraction of aberrant junctions in ALS and FTD have TDP‐43 binding sites nearby (Figure 3I). Interestingly, AGO2 and UPF1 show higher binding enrichment near aberrant splicing sites than TDP‐43, suggesting these aberrant RNAs may undergo degradation through AGO2‐miRNA [39] and UPF1 [40] mediated pathway.

In addition, we analyzed published TDP‐43 knockdown datasets of human cell lines (Table S2) and found that enriched biological process (BP) terms of genes with up‐regulated unannotated junctions in cell lines (Figure S3I) are similar to the BP terms of genes containing aberrant splicing junctions we identified in patients’ frontal cortex, both of which are related to GTPase mediated signal transduction and neuron projection in ALS (Figure 3J) and FTD (Figure 3K). GTPase signaling, particularly involving the Rho family, is closely linked to neurodegenerative diseases, with its dysregulation contributing to neuronal damage and neuroinflammation [41]. Moreover, more than 40% of TDP‐43 targets identified in human cell lines have aberrant splicing junctions in ALS/FTD patients (Figure 3L). In summary, the occurrence of aberrant junctions in ALS and FTD patients is closely related to TDP‐43 pathology and regulates different cell types between ALS (glial cells, especially oligodendrocytes) and FTD (neurons).

### 2.5 Neuron or Oligodendrocyte Specific Genes Harboring Aberrant Splicing Junctions Are Potential Disease Markers

**FIGURE 4:**
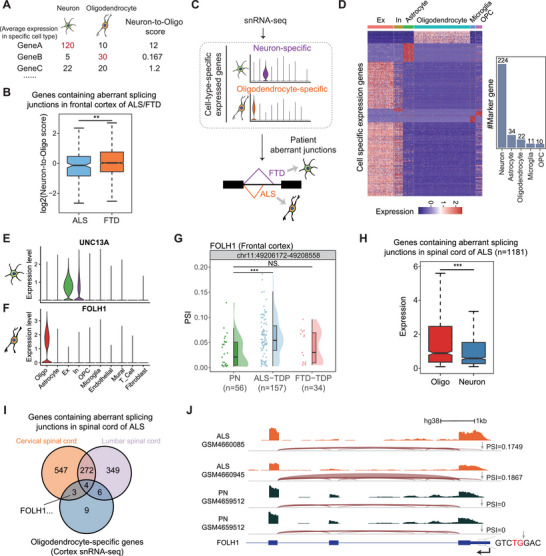
Identification of potential disease biomarkers in neuron‐ or oligodendrocyte‐specific genes. (A) Diagram comparing the contribution of neurons and oligodendrocytes by calculating Neuron‐to‐Oligo score based on snRNA‐seq cell type average expression. (B) Box plot showing Neuron‐to‐Oligo score distribution of genes containing aberrant junctions in the frontal cortex of ALS‐TDP and FTD‐TDP patients. (C) Diagram of disease biomarkers identified in cell‐type‐specific expressed genes. (D) Heatmap showing expression of identified cell‐type‐specific expressed genes in each cell type (left panel), and bar plot showing the number of marker genes of each cell type (right panel). (E‐F) Single‐cell gene expression violin plots of UNC13A (E) and FOLH1 (F). (G) Scatter and violin plots illustrating PSI changes of the aberrant junction in FOLH1 among different sample groups in the frontal cortex. (H) Box plot showing the neurons’ and oligodendrocytes’ expression distribution of genes containing aberrant splicing junctions in the spinal cord of ALS patients. (I) Venn plot depicting the intersection of genes containing aberrant junctions detected in the cervical spinal cord, lumbar spinal cord of ALS‐TDP patients, and oligodendrocyte‐specific genes. (J) Splicing tracks of the aberrant junction in FOLH1 between ALS patients and PN in the frontal cortex. Grey arrows indicate the cryptic splice site. The *p*‐values were calculated by the Wilcoxon test.

Since we found that TDP‐43‐regulated aberrant splicing junctions in ALS/FTD seemed to occur in different cell types, we further utilized snRNA‐seq data to calculate the average expression levels across all neurons and oligodendrocytes in PN cortex samples. This allowed us to compare their respective contributions for a given gene more comprehensively by calculating the Neuron‐to‐Oligo score, relative expression in neurons over oligodendrocytes (Figure 4A). The distribution of Neuron‐to‐Oligo score in genes containing aberrant junctions in the frontal cortex of ALS and FTD, respectively, revealed that oligodendrocytes predominantly contributed to aberrant junctions in ALS, whereas neurons played a more significant role in FTD (Figure 4B). We sought to further identify marker genes for the two diseases based on this phenomenon. Briefly, we identified ALS/FTD markers based on cell‐type‐specific expressed genes and disease‐specific aberrant splicing genes. For instance, genes specifically expressed in neurons, with their aberrant junctions only detected significantly up‐regulated in FTD patients, would be identified as biomarkers of FTD. Conversely, genes specifically expressed in oligodendrocytes with their aberrant junctions only detected significantly up‐regulated in ALS patients would be identified as biomarkers of ALS (Figure 4C).

We applied a rigorous criterion to identify genes that exhibit specific expression patterns in each cell type using the Seurat R package. Among them, we identified 224 genes that were exclusively expressed in neurons and 22 genes that were specifically expressed in oligodendrocytes, as candidate genes of cell‐type‐specific biomarkers (Figure 4D), as exemplified by the canonical marker genes for each cell type (Figure S4A,B). In these cell‐type‐specific expression genes, we identified a series of disease‐ and cell‐type‐specific splicing events. Among them, *UNC13A* [36, 37] and *STMN2* [42], which were previously widely reported as TDP‐43 target genes, were specifically expressed in neurons (Figure 4E; Figure S4C). Their corresponding aberrant junctions were detected in FTD in the frontal cortex but were not significantly changed in ALS (Figure 3F; Figure S4D), further indicating the differences between ALS and FTD in cell types affected by TDP‐43 pathology in the patients’ cortex. The reason why FTD patients exhibited more pronounced alterations in splicing (Figure 3G) and expression (Figure S1D,E) in the cortex compared to ALS could be attributed to the fact that damaged neurons in FTD possess a greater diversity of cell types and a larger repertoire of specific genes than glial cells do.

Interestingly, we identified some novel cell‐type‐specific expressed target genes whose splicing junction change appeared in a disease‐specific manner in ALS/FTD. *FOLH1* was specifically expressed in oligodendrocytes (Figure 4F), and the aberrant splicing junction of *FOLH1* showed significantly up‐regulated in ALS patients’ frontal cortex, but not in FTD (Figure 4G). Mouse models of *Folh1* knockout showed significant disruption of myelin and axons in previous studies [43], which was consistent with what we observed in ALS patients. In addition, *RBFOX3* was a canonical neuron cell marker that was specifically expressed in neurons (Figure S4E). The aberrant splicing junction of *RBFOX3* showed significantly up‐regulated in FTD in the frontal cortex, but not in ALS (Figure S4F). While ALS was known to affect both upper and lower motor neurons [44], we further explored whether splicing changes observed within the frontal cortex would occur in ALS patients’ spinal cord. In the ALS spinal cord, aberrant junctions were also more contributed by oligodendrocytes than neurons (Figure 4H). Surprisingly, 13 of 22 oligodendrocytes specifically expressed genes that were detected to harbor aberrant junctions in the spinal cord, including *FOLH1*, which was also detected in the frontal cortex (Figure 4I; Figure S4G). In ALS patients, *FOLH1* utilized an abnormal 5′ splice site within the 3′UTR that was rarely utilized in PN samples (Figure 4J). Enriched GO‐terms of the genes containing aberrant junctions in the spinal cord showed that signal transmission is affected (Figure S4H), which is related to the function of oligodendrocytes [45].

### 2.6 A Robust Random Forest Model Validates a Cell‐Type‐Specific Splicing Signature for Distinguishing ALS and FTD

**FIGURE 5:**
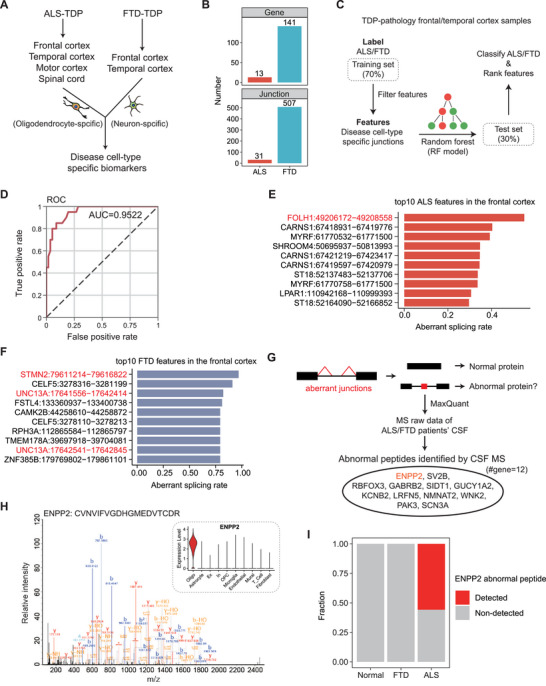
Construction of a Random Forest model to classify ALS/FTD patients and identification of abnormal peptides. (A) Diagram of identifying disease cell‐type‐specific biomarkers. (B) Bar plots showing the number of identified cell‐type‐specific disease biomarkers at gene and junction levels, respectively. (C) Diagram of constructing RF model to classify ALS and FTD cortex samples. (D) ROC curves of the RF models using cell‐type‐specific biomarkers as features. (E‐F) Bar plots showing the top 10 ALS‐specific (E) or FTD‐specific (F) biomarkers with the highest rate in the frontal cortex samples of patients. (G) Diagram of searching aberrant junctions caused abnormal peptides in ALS/FTD patients’ CSF MS data. (H) MS/MS spectrum of an abnormal peptide of ENPP2 detected in patients’ CSF, and the violin plot of expression pattern of ENPP2 at the single‐cell level. (I) Bar plot showing the proportion of samples with detected abnormal ENPP2 peptides in ALS, FTD, and normal control groups.

Since numerous biomarkers had been identified, we aimed to ascertain whether these cell‐type‐specific biomarkers are sufficient for distinguishing ALS and FTD patients’ cortex with TDP‐43 inclusion and determining the key features that play a crucial role in differentiating between the two patient groups. To further expand potential biomarkers, we employed the approach mentioned above (Figure 4C) to identify cell‐type‐specific biomarkers in the frontal cortex, temporal cortex, motor cortex, and spinal cord of ALS‐TDP patients, as well as in the frontal cortex and temporal cortex of FTD‐TDP patients (Figure 5A). Due to the pronounced significant tissue specificity of the cerebellum in ALS [46], we also observed that the splicing pattern of the cerebellum in PCA coordinates was substantially different from the cortex and spinal cord on PC1 (Figure S5A), and thus splicing change in this region was not taken into consideration here. After excluding cerebellum samples, we identified 31 oligodendrocyte‐specific junctions belonging to 13 genes in ALS and 507 neuron‐specific junctions belonging to 141 genes in FTD (Figure 5B; Table S7). Next, we utilized cell‐type‐specific junctions identified in two diseases as features and employed a random forest (RF) model to classify cortex samples of ALS‐TDP and FTD‐TDP patients to test the classification efficacy. To avoid information leakage and overfitting, we partitioned the data into 70% for training and 30% for testing. Importantly, cell‐specific disease markers used as model features were chosen solely based on the training set data (Figure 5C). We found that these cell‐type‐specific biomarkers exhibit excellent classification results when classifying cortex samples of ALS and FTD (Figure 5D). We also applied k‐fold (five‐fold) cross‐validation, performing feature selection and model training on each fold independently to test the model performance. Notably, these biomarkers demonstrated robust classification accuracy under more stringent validation (Figure S5B). This demonstrated that the biomarkers we identified are largely adequate for discriminating between ALS and FTD, further confirming the differences in affected cell populations within the cortex of ALS and FTD patients.

Furthermore, for each cell‐type‐specific aberrant splicing junction, we calculated the rate of patient samples with PSI values above the 75^th^ percentile of the normal control distribution. Aberrant splicing of *FOLH1*, as described above (Figure 4J), showed the highest rate among oligodendrocyte‐specific ALS biomarkers (Figure 5E). Among FTD biomarkers, aberrant *STMN2* splicing showed the highest rate (Figure 5F), consistent with its previously reported association with FTD‐TDP pathology [42]. Taken together, a higher rate of aberrant splicing in patients might suggest greater potential for reliable detection of disease‐associated aberrant splicing junctions.

### 2.7 Aberrant Splicing Transcripts Generated Abnormal Peptides in Patients’ CSF

Furthermore, we hypothesized that these aberrant junctions might produce abnormal peptides and subsequently utilized MaxQuant [47] program to search ALS/FTD patients’ cerebrospinal fluid (CSF) mass spectrometry data by focusing on peptides generated due to aberrant splicing events (Figure 5G). We found that 12 genes containing aberrant junctions could generate abnormal peptides detectable in CSF (Table S3). However, most aberrant splicing events were not detected within CSF, possibly because transcripts containing aberrant splicing junctions underwent degradation via the NMD pathway or the peptides were not secreted into CSF. As an example, we detected the peptides generated by an oligodendrocyte‐specific aberrant splicing junction in *ENPP2* (Figure 5H). This aberrant junction was significantly up‐regulated in lumbar spinal cords of ALS patients (Figure S5E,F). Moreover, it was detected in 56% ALS patients’ CSF samples but not in FTD or control samples, suggesting its potential as an ALS‐specific biomarker of oligodendrocyte damage (Figure 5I). In summary, the detection of peptides from oligodendrocyte or neuron‐specific aberrant junctions in patients’ CSF further demonstrated the potential of using them as biomarkers for clinical detection.

### 2.8 Validation of Identified Targets in Independent Datasets and Patient Samples

**FIGURE 6:**
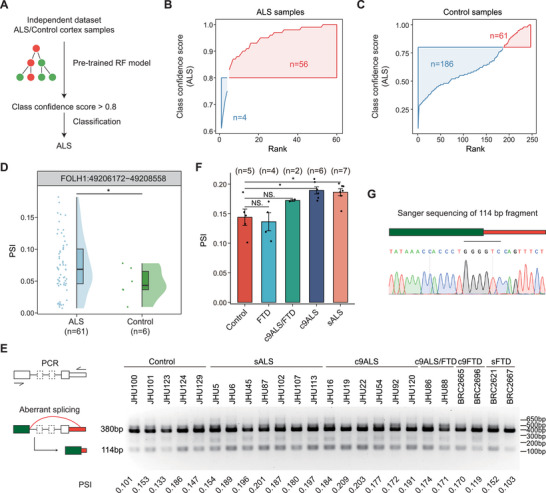
Model testing on an independent dataset and validation of the ALS oligodendrocyte‐specific target in patient tissues. (A) Diagram of testing the pre‐trained RF model on an independent dataset. (B‐C) Cumulative curve of negative class (ALS) confidence score of frontal cortex ALS samples (B) and PN control samples (C). (D) Scatter and violin plots illustrating PSI changes of aberrant junctions in FOLH1 among the two sample groups in the frontal cortex. (E) RT‐PCR validation of FOLH1 splicing change in human postmortem frontal cortex. The RT‐PCR target of the aberrant splicing event has 114 bp. (F) Quantification of the RT–PCR products in E from FOLH1 aberrant splicing junction. PSI represents the percent of spliced‐in of the aberrant junction. Error bars represent the 95% confidence interval of the mean. (G) Sanger sequencing confirming the aberrant splicing junction.

To further validate the generalizability of our biomarker strategy, we applied the trained classification model to an independent dataset, GSE124439, which contains frontal cortex samples from 60 ALS patients. Samples with an ALS classification confidence score greater than 0.8 were considered to be predicted as ALS (Figure 6A). Using our pre‐trained random forest model, 56 out of 60 samples were correctly classified as ALS with a confidence score above 0.8, achieving an accuracy of 93.3% on the independent dataset (Figure 6B). In contrast, for the PN control samples, the majority of classification scores fell between 0.2 and 0.8, suggesting that most PN samples could not be confidently classified as either ALS or FTD (Figure 6C). These findings further supported the robustness and discriminative power of the classification biomarkers we identified. Additionally, we found that some of the ALS cell‐type‐specific targets identified as biomarkers in the above data were also validated in this independent dataset, such as the aberrant junction in the *FOLH1* gene (Figures 6D and 4J).

While neuron‐specific aberrant splicing events, such as those affecting *U* *NC13A* [36, 37] and *STMN2* [42], have been previously validated in ALS/FTD patients, oligodendrocyte‐specific splicing alterations remain largely unconfirmed. To address this gap, we focused on validating oligodendrocyte‐specific splicing changes by directly assessing the aberrant splicing junction of *FOLH1* using RNA extracted from the frontal cortex of ALS/FTD patients, thereby providing additional support for the RNA‐seq findings. RTPCR primers were designed to target the aberrant junction identified in RNA‐seq, and RT‐PCR was subsequently performed on the frontal cortex samples. The 114 bp aberrant splice band, corresponding to the target splicing junction, was observed with higher intensity in ALS patient samples compared to controls (Figure 6E). As expected, we did not observe a notable increase in aberrant *FOLH1* splicing in FTD patients, including two C9orf72‐associated cases, two sporadic cases, and two individuals with combined ALS and FTD, compared with controls. Quantification of the target band relative to the total splice products was used to calculate the PSI (Percent spliced in) values, which revealed that the aberrant splicing junction was significantly elevated in both C9orf72‐associated (*n* = 6) and sporadic ALS (*n* = 7) patients compared to healthy controls (*n* = 5), but not in FTD patients (*n* = 4) (Figure 6F). To further validate that the targeted band corresponded to the splicing junction identified in RNA‐seq, the 114 bp band was excised and subjected to Sanger sequencing. The sequencing confirmed the presence of the expected splicing junction, consistent with the RNA‐seq results (Figure 6G), thereby supporting the accuracy of data analysis and confirming the up‐regulation of this aberrant splicing junction in ALS patients.

### 2.9 Cell‐Type‐Specific Regulation of Shared Genes Under TDP‐43 Depletion

**FIGURE 7:**
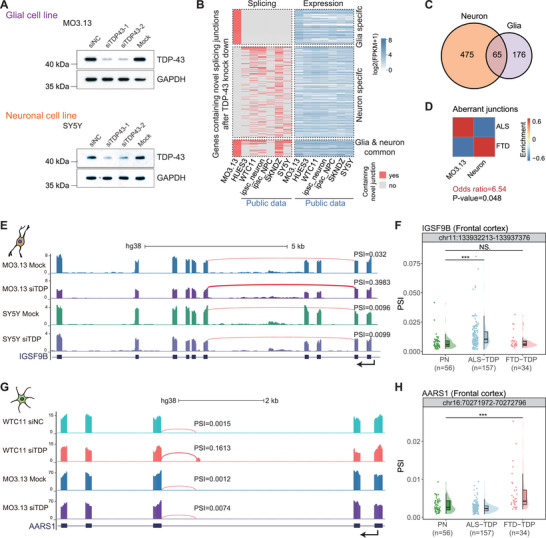
Genes shared by glial and neuronal cell lines show differential regulation by TDP‐43. (A) Western blot results showing TDP‐43 knockdown effect at the protein level in MO3.13 and SY5Y. (B) Heatmap showing genes containing novel junctions or not (left panel) and expression level (right panel) in each cell line. Red boxes indicated genes in the corresponding cell line containing up‐regulated novel splicing junctions after TDP‐43 knockdown; gray box indicated not. The genes were divided into three groups, from top to bottom, which were genes with novel junctions only in the glia cell line, only in neuron cell lines, and in both glia and neuron cell lines, respectively. (C) Venn plot depicting the intersection of genes containing up‐regulated novel splicing junctions after TDP‐43 knockdown identified in neuron cell lines and the glial cell line. (D) Heatmap showing the enrichment of aberrant junction activation in the frontal and temporal cortex of ALS‐TDP/FTD‐TDP patients in MO3.13 and neuron cell lines. Odds ratio and *p*‐value are calculated by Fisher's exact test. The contingency table is constructed on the overlap number between up‐regulated aberrant junctions in patients and up‐regulated novel splicing junctions after TDP‐43 knockdown in cell lines. (E) UCSC genome browser view of normalized RNA‐seq signals of IGSF9B. The quantified PSI values of the targeted junction are labeled for all samples, respectively. (F) Scatter and violin plots illustrating PSI changes of aberrant junctions in IGSF9B among different sample groups in the frontal cortex. (G‐H) Same as E‐F for gene AARS1, respectively. The *p*‐values were calculated by the Wilcoxon test.

Although the same gene is expressed in different cell types, it might still be subject to distinct regulation. Thus, in addition to cell‐type‐specifically expressed genes, we further explored cell‐type‐specific splicing events in response to TDP‐43 dysfunction across neuronal and glial cell lines. Due to the current lack of TDP‐43 dysfunction data in oligodendrocytes, we performed RNA‐seq in the oligodendrocyte cell line MO3.13 and neuron cell line SY5Y under TDP‐43 knockdown, respectively. The knockdown effect at both protein and RNA levels is efficient (Figure 7A; Figure S6A), and data reproducibility is high (Figure S6B). In addition, we collected public RNA‐seq data of HUES3, WTC11, SKNDZ, iPSC‐derived neurons, and neural progenitor cells (NPC) cell lines under TDP‐43 knockdown (Table S2). Upon calculating the novel junctions that exhibited significant up‐regulation in each cell line following TDP‐43 knockdown, we observed a small number of junctions jointly regulated by TDP‐43 in both neuronal and glial cell lines. Most genes containing these junctions were expressed in both cell line types, but strangely exhibited differential splicing regulation under TDP‐43 knockdown (Figure 7B). The analysis of the gene sets regulated by TDP‐43 across all neuronal cell lines also revealed a limited overlap with those in the glial cell line (Figure 7C).

Interestingly, enrichment analysis of aberrant splicing junctions detected in the frontal cortex of ALS/FTD patients revealed that the up‐regulated novel junctions induced by TDP‐43 knockdown were preferentially enriched in the glial cell line for ALS and in neuronal cell lines for FTD (Figure 7D). This correspondence indicates that the in vitro models partially recapitulate the cell‐type‐specific splicing patterns present in patients. As exemplar events, an exon skipping event in *IGSF9B* and another event in *MATCAP1* were specifically observed in the MO3.13 cell line and ALS patients’ frontal cortex, while they were not significantly up‐regulated in neuron cell lines and FTD patients (Figure 7E,F; Figure S6C,D). The same junction in *IGSF9B* was detected in the motor cortex of ALS‐TDP (Figure S6E), suggesting that upper motor neuron lesions in ALS may also be related to oligodendrocyte involvement. Correspondingly, aberrant splicing junctions in *AARS1* and *KALRN* specifically detected in neuron cell lines were also identified in FTD patients (Figure 7G,H; Figure S6F,G).

### 2.10 TDP‐43 Dysfunction Affects Cell‐Type‐Specific Splicing Depending on the Cellular Context

**FIGURE 8:**
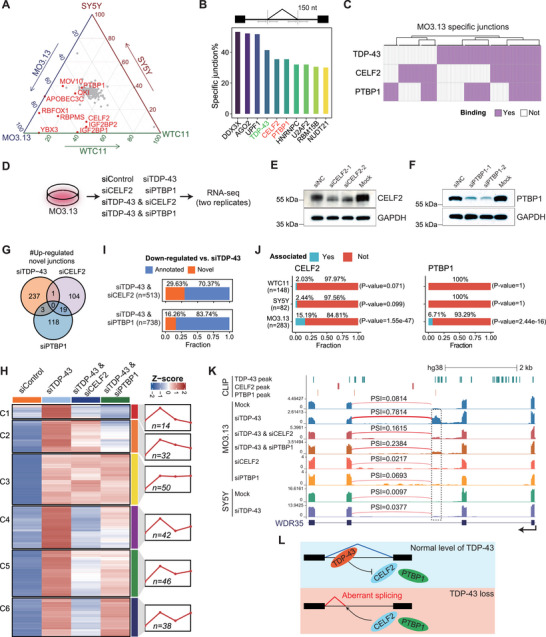
TDP‐43 dysfunction affected splicing in a cell‐type‐specific way mediated by other RBPs. (A) Ternary diagram showing RBPs expression difference among SY5Y, WTC11, and MO3.13. RBPs highly expressed in MO3.13 are marked in red, having at least one set of log2(fold change) greater than 2.5 compared to SY5Y or WTC11. (B) The top 10 percent of MO3.13 specifically up‐regulated junctions bound by RBPs in ±150 nt region flanking the splice sites. (C) Heatmap showing TDP‐43, CELF2, and PTBP1 binding within ±150 nt region flanking the splice sites of MO3.13, specifically up‐regulated junctions based on CLIP‐seq datasets. (D) Diagram of RNA‐seq settings on the MO3.13 cell line. (E‐F) Western blot results showing CELF2 (E) and PTBP1 (F) knockdown effects at the protein level. (G) Venn plot depicting the intersection of up‐regulated novel splicing junctions identified in MO3.13 following individual knockdown of TDP‐43, CELF2, or PTBP1. (H) K‐means clustered heatmap showing PSI changes of TDP‐43‐regulated novel junctions when CELF2 or PTBP1 is depleted under TDP‐43 deficient. (I) Percentage of down‐regulated novel and annotated junctions upon CELF2 or PTBP1 knockdown under TDP‐43 knockdown condition in MO3.13. (J) Bar plot showing the fraction of CELF2‐associated junctions (left panel) or PTBP1‐associated junctions (right panel) among TDP‐43‐regulated novel junctions in WTC11, SY5Y, and MO3.13 cell lines. The *p*‐values were calculated using Fisher's exact test to assess the significance of overlap. (K) UCSC genome browser view of normalized RNA‐seq signals of WDR35. CLIP‐seq peaks of TDP‐43, CELF2, and PTBP1from the POSTAR3 database are shown. The quantified PSI values of the targeted junction are labeled for all samples, respectively. (L) Schematic showing how CELF2 and PTBP1 function in regulating RNA aberrant splicing in the context of TDP‐43 loss.

Given the observation that TDP‐43 knockdown led to cell‐type‐specific splicing events, independent of target gene expression, we hypothesized that other RBPs (RNA‐binding proteins) might regulate this process as cofactors. Thus, we performed clustering analysis based on RBP expression across human cell lines. Notably, non‐neuronal cell lines, neuroblastoma cell lines, the iPSC‐derived NPC cell line, and iPSC‐derived neuron cell lines formed four distinct clusters (Figure S7A), indicating that the expression of RBPs largely determined the specificity of cells. Subsequently, three representative cell lines were selected for further investigation: MO3.13 representing oligodendrocytes, SY5Y representing neuron cells, and WTC11 representing iPSC‐derived neurons. Interestingly, there was only a small overlap in genes containing novel splicing junctions responding to TDP‐43 loss in the above three cell lines (Figure S7B). Then, we calculated the differential expression of RBPs among these three cell lines with a particular focus on those highly expressed in MO3.13, including 10 RBPs highlighted in red (Figure 8A).

Cell context shapes the splicing events under TDP‐43 knockdown [48], including the involvement of other RBPs. Statistics of the binding ratio of each RBP near the splicing junctions specifically responding to TDP‐43 knockdown in MO3.13 revealed that CELF2 and PTBP1 are bound near a large number of specific junctions (within flanking 150 nt), and their binding ratios are similar to TDP‐43 (Figure 8B). Although AGO2 and UPF1 showed higher binding ratios, their enrichment likely reflected transcript degradation rather than direct involvement in splicing. In contrast, CELF2 and PTBP1, as splicing‐related RBPs, exhibited high binding ratios at both MO3.13‐specific TDP‐43 responsive junctions (Figure 8B) and also at patient‐derived aberrant splicing junctions (Figure 3I), indicating their key role in mediating cell‐type‐specific splicing events and disease pathogenesis.

Meanwhile, the expressions of *CELF2* and *PTBP1* are higher in MO3.13 than in the other two cell lines (Figure S7C,D). Examining each MO3.13‐specific TDP‐43‐regulated junction individually, we found that most of the junctions bound by TDP‐43 are also bound by at least one of CELF2 and PTBP1 (Figure 8C). To explore whether these two RBPs regulate abnormal splicing under TDP‐43 depletion, we performed six sets of RNA‐seq, including negative controls (NC), TDP‐43 knockdown, CELF2 knockdown, PTBP1 knockdown, TDP‐43 and CELF2 double knockdown, as well as TDP‐43 and PTBP1 double knockdown in MO3.13 cells with two replicates for each condition (Figure 8D). The knockdown is efficient at the RNA level (Figure S7E), and the two replicates of RNA‐seq show good reproducibility (Figure S7F). The Western blot also showed that the knockdown effects of PTBP1 and CELF2 are efficient compared to the NC (Figure 8E,F).

We found that knockdown of either CELF2 or PTBP1 alone also caused the up‐regulation of a subset of novel junctions, consistent with their roles as splicing regulators, but the overlap of which with TDP‐43‐regulated novel junctions was minimal (Figure 8G). This suggested that, at a normal level of TDP‐43, CELF2 and PTBP1 each influenced splicing but largely regulated events distinct from those controlled by TDP‐43. However, when we examined the effects of depleting these RBPs together with TDP‐43, we observed a pronounced impact on TDP‐43 induced aberrant splicing. We investigated the changes in TDP‐43‐regulated novel junctions under TDP‐43 and CELF2 or PTBP1 knockdown simultaneously. The results showed that, except for 50 junctions belonging to the C3 cluster that are not changed observably after CELF2 or PTBP1 knockdown, other junctions have obviously attenuated responses to TDP‐43 after knocking down CELF2 or PTBP1 (Figure 8H), and there are more junctions down‐regulated when knocking down CELF2 than PTBP1. Nearly 30% of the junctions down‐regulated under TDP‐43 and CELF2 double knockdown, compared to only TDP‐43 knockdown, are novel junctions, and the fraction is 16% under TDP‐43 and PTBP1 double knockdown (Figure 8I), indicating that a considerable part of novel splicing events caused by TDP‐43 deletion are probably promoted by CELF2 and PTBP1, especially CELF2. Moreover, among the down‐regulated novel junctions under TDP‐43 and CELF2 double knockdown, there are 43 (15.19%) junctions repressed by TDP‐43 in MO3.13, showing significant overlap with up‐regulated junctions under TDP‐43 depletion in MO3.13, obviously more than those repressed by TDP‐43 in WTC11 and SY5Y, where CELF2 has a lower expression. Meanwhile, 19 (6.71%) of the down‐regulated novel junctions under TDP‐43 and PTBP1 double knockdown junctions repressed by TDP‐43 in MO3.13, showing significant overlap with up‐regulated junctions under TDP‐43 depletion in MO3.13, but showing no overlap with those repressed by TDP‐43 in WTC11 and SY5Y (Figure 8J; Figure S7G).

TDP‐43 binding near the novel junctions of *WDR35* was associated with the significant inclusion of a cryptic exon upon TDP‐43 depletion in MO3.13, but not in SY5Y, indicating a cell‐type‐specific splicing dysregulation. The cryptic exon was significantly reduced after knocking down both TDP‐43 and CELF2 or PTBP1, whereas single knockdown of CELF2 or PTBP1 in the presence of TDP‐43 did not lead to cryptic splicing (Figure 8K). The cryptic exon in *DENND2B* showed a similar pattern and was significantly reduced after knocking down CELF2 or PTBP1 under TDP‐43 depletion (Figure S7H). More importantly, we observed that some aberrant splicing events specifically occurred in ALS patients and were also detected in MO3.13 after TDP‐43 knockdown, and were significantly attenuated after both TDP‐43 and CELF2 or PTBP1 knockdown, such as the exon skipping event in *IGSF9B* that was significantly up‐regulated in the frontal cortex of ALS patients (Figure 7E,F; Figures S7I). Notably, both CELF2 and PTBP1 showed significantly altered expression in patient samples compared with normal controls (Figure S7J), further indicating their involvement in disease‐associated splicing dysregulation. Together, these findings suggested that the promotion of abnormal splicing by other RBPs likely played an important role in the pathogenesis of ALS/FTD.

Based on these findings, we proposed a model in which, under normal conditions, TDP‐43 binds near aberrant splice sites to prevent the recruitment of other RBPs and suppress abnormal splicing. When TDP‐43 was depleted, CELF2 or PTBP1 could instead bind near these sites and promote the use of aberrant splicing junctions (Figure 8L). In summary, TDP‐43 may compete with other RBPs for binding near aberrant splicing sites, and the occurrence of abnormal splicing in the absence of TDP‐43 is mediated by other RBPs, which leads to cell‐type‐specific regulation.

## 3 Discussion

In this study, we uncovered aberrant splicing junctions mediated by TDP‐43 pathology that occurred within distinct cell types in ALS and FTD, indicating a potentially different pathogenic mechanism between the two diseases, and we identified bona fide disease biomarkers of ALS and FTD, respectively, at a cell‐type‐specific level for the first time.

In ALS patients’ cortex, neuronal genes appeared up‐regulated, whereas in FTD they were down‐regulated. However, these apparent differences largely reflected shifts in cell‐type composition rather than genuine transcriptional alterations. Deconvolution analysis (Figure 1I) and pseudo‐bulk validation (Figure S8) confirmed that oligodendrocyte loss in ALS elevated neuronal gene expression at the bulk level as an artifact of altered cell composition, while single‐cell data showed no corresponding change. This previously overlooked confounding factor has likely influenced the interpretation of earlier transcriptomic studies of ALS and FTD.

Building on the established role of TDP‐43 in splicing regulation [49, 50, 51], we found that aberrant splicing junctions predominantly occurred within oligodendrocytes in ALS patients’ cortex and within neurons in FTD patients’ cortex. To further validate the distinct involvement of oligodendrocytes in ALS and neurons in FTD, we identified cell‐type‐specific aberrant splicing biomarkers for ALS and FTD through integrating bulk RNA‐seq with snRNA‐seq. These biomarkers not only supported the differential cell‐type vulnerability but also demonstrated strong classification performance even in independent datasets. Future studies incorporating direct pTDP‐43 quantification will help clarify how TDP‐43 pathology burden relates to splicing alterations across cortical regions. In addition, mechanistic analyses revealed that RBPs such as CELF2 and PTBP1 may further promote aberrant splicing under TDP‐43 loss, underscoring a complex regulatory network underlying cell‐type‐specific vulnerability.

Our findings fill some gaps in the field and have important clinical implications. Differing from most previous studies, we mainly focused on the possible differences in the pathogenic mechanisms between ALS and FTD, and revealed molecular differences between ALS and FTD based on high‐throughput sequencing data. We have revealed distinct TDP‐43‐affected cell types in ALS and FTD, and uncovered that ALS patients had oligodendrocyte involvement in the frontotemporal cortex, which was previously more concerned in FTD diseases. Early TDP‐43 aggregation and pathological features resulting from TDP‐43 pathology, including abnormal splicing of *STMN2*, could be detected before clinical features appeared [52]. Thus, these cell‐type‐specific disease biomarkers provided new clues for early diagnosis. Notably, several aberrant spliced transcripts produced detectable peptides in CSF, such as the oligodendrocyte‐specific aberrant splicing junction in *ENPP2*, further highlighting the potential of these biomarkers as diagnostic indicators. As TDP‐43‐induced abnormal splicing events were cytotoxic, these aberrant splicing junctions might also serve as therapeutic targets, where blocking pathogenic splicing could have disease‐modifying effects.

Our observations are well supported by prior ALS/FTD studies. Oligodendrocyte cytoplasmic TDP‐43 inclusions were reported to be common in affected cortex samples of ALS/TDP patients [53], and dysfunction of oligodendrocytes had been detected in the motor cortex and spinal cord of ALS patients, as well as in ALS mouse models, which strongly accelerates disease progression [54]. Genomic [16] and transcriptomic [17] analyses also pointed to oligodendrocyte vulnerability in ALS.

For FTD patients, neuron loss has been confirmed to intensify with the progression of TDP‐43 pathology [55]. However, some clues show neuronal involvement may be more severe in FTD than in ALS within the same cortical regions. Cryptic exons in neuron‐specific genes such as *STMN2* and *UNC13A* are detected at much higher levels in FTD cortices than in ALS, and truncated *STMN2* has been identified as a biomarker of TDP‐43 pathology in FTD [42]. Moreover, it has been reported that truncated *STMN2* was detected more in excitatory neurons in both frontal and occipital cortex in FTD than in ALS patients at single cell level [12]. Our findings here shed light on why aberrant splicing of these two genes in the cortex was more pronounced in FTD than in ALS patients, a question that was previously unanswered. Furthermore, our re‐analysis of this single‐cell dataset [12] also revealed disease‐associated shifts in cell‐type composition, with reduced oligodendrocytes in the frontal cortex of ALS patients and decreased neuronal populations in FTD (Figure S8D).

In our analysis, *STMN2* exhibited clear aberrant splicing in the spinal cord of ALS‐TDP patients but not in the frontotemporal cortex (Figure S9A). In contrast, *UNC13A* mis‐splicing was minimal in both the frontotemporal cortex and the spinal cord of ALS patients (Figure S9B), despite its known association with ALS. Although the severe motor neuron loss in ALS spinal cord could reduce the detectability of splicing defects, *UNC13A* mis‐splicing remained largely absent even in samples with robust *STMN2* defects, indicating that neuron loss alone is unlikely to explain this pattern. Previous studies have indicated that, unlike *STMN2*, *UNC13A* transcripts harbor a premature termination codon (PTC) that triggers NMD (36), leading to degradation in affected neurons. This mechanism, together with potential tissue‐specific differences in NMD efficiency, may account for the reduced detectability of *UNC13A* mis‐splicing in spinal cord tissue relative to cortical regions. Therefore, it is important to note that the lack of pronounced *UNC13A* mis‐splicing in spinal cord tissue may reflect both disease stage and tissue‐specific vulnerability.

We also observed that *NPTX2*, a recently reported novel therapeutic target for neurodegenerative diseases whose overexpression would result in cytotoxicity [56], was up‐regulated in neurons of FTD patients based on snRNA‐seq data but down‐regulated in bulk RNA‐seq data, indicating that neurotoxic pathways were activated and led to neuronal death in FTD patients. Although this phenomenon has been implied in previous studies, the cause of neuronal or glial cell loss and the relationship between cell loss and TDP‐43 pathology remain unclear. Here, we reveal that this specific cell loss is highly related to aberrant splicing junctions that might be caused by nuclear loss of TDP‐43 and promoted by other RBPs, providing new targets and ideas for future disease diagnosis and treatment. In addition, part of the cell‐type compositional changes we observed may also reflect secondary reactive processes, particularly gliosis. In FTD, neuronal loss in the frontal cortex is expected to induce reactive astrogliosis and microgliosis, which could partially contribute to the increased relative representation of glial cells.

Notably, to our knowledge, there are no reports on oligodendrocyte‐specific gene abnormalities in ALS/FTD so far; the oligodendrocyte‐specific biomarkers we identified can effectively classify ALS and FTD patients. Moreover, we validated the aberrant splicing of an oligodendrocyte‐specific gene, *FOLH1*, in patient brain tissues, further supporting the cell‐type specificity and clinical relevance of our findings.

However, our study still has some limitations. First, in consideration of clinical and pathological heterogeneities being recognized features of neurodegenerative diseases, our analysis demonstrated statistical differences, but not all individuals conformed to what we observed. Thus, the specific factors that determine individual differences still require further studies to better identify biomarkers of the two diseases. Moreover, we assessed whether “ALS‐high” and “ALS‐low” subgroups show clinical or genetic distinctions. C9orf72‐positive cases were more frequent in the “ALS‐low” group, and “ALS‐low” patients showed trends toward earlier onset, earlier death, and longer disease duration, although none reached statistical significance due to limited sample size and substantial heterogeneity. These findings underscore the need for larger, well‐annotated cohorts to clarify potential subgroup differences and further strengthen biomarker development.

Due to certain differences between cell lines and in vivo environments, it may not be realistic to reflect the in vivo situation. Although MO3.13 cells provided a useful glial context for establishing RBP‐dependent splicing mechanisms, we speculate that iPSC‐derived oligodendrocytes might serve as an alternative and potentially more physiologically relevant model for future studies. Such systems may better recapitulate oligodendrocyte maturation and will be valuable for validating cell‐type‐specific responses to TDP‐43 pathology in follow‐up work.

Furthermore, since many patients were diagnosed with both ALS and FTD, whether there is a causal relationship between oligodendrocyte and neuron involvement also deserves further study. For example, 5%‐25% of ALS patients had behavioral alterations along with the course of the disease, leading to the diagnosis of FTD [57]. Whether such symptoms arise from oligodendrocyte dysfunction affecting neuronal integrity in the frontotemporal cortex remains unclear. Notably, in our validation of the aberrant splicing event in *FOLH1*, we observed that its increase relative to controls was negligible in patients diagnosed with both ALS and FTD. However, due to the limited number of samples in this subgroup, these findings may not reflect a generalizable trend. Further validation in larger cohorts and across additional targets is needed to elucidate consistent patterns and potential mechanistic links. For future studies, a research system that can more truly restore the in vivo environment is required; for instance, a human neural network model called iNets has been developed to simulate disease situations in which neuronal and glial maturation is similar to that of the human cortex [56]. In addition, due to the limited size of data we used, especially the number of CSF mass spectrometry samples, we may not have identified all the potential targets, and since we identified biomarkers based on patient postmortem samples, the real performance of the set of biomarkers in patients’ clinically accessible tissues needs to be further studied. Here, we identified *ENPP2* as a specific target to indicate oligodendrocyte damage that could be detected in patients’ CSF. We proposed that with the increased patient sample sizes and improved sensitivity in detection methods, more low‐abundance abnormal peptides would be identified as targets for the diagnosis and treatment of ALS/FTD.

In summary, this work delineates distinct molecular mechanisms in ALS and FTD, emphasizing that TDP‐43 pathology results in aberrant splicing, accompanied by oligodendrocyte loss in ALS and neuron loss in FTD, and additionally provides potential cell‐type‐specific biomarkers for these diseases. Deciphering these molecular differences deepens understanding of pathogenic pathways and paves the way for improved clinical strategies.

## 4 Materials and Methods

### 4.1 Cell Culture

Both MO3.13 (BNCC, BNCC360263) and SY5Y (BNCC, BNCC100158) cells were cultured in DMEM (Gibco) medium supplemented with 10% FBS in a humidified incubator at 37°C with 5% CO_2_.

### 4.2 siRNA Transfection

For knockdown TDP‐43, CELF2, and PTBP1 in MO3.13, 1 µL siRNA (JTSBIO Co., Ltd., Wuhan, China) was added into one well of 12‐well plates with 2 µL RNAiMAX (Invitogen) according to manufacturers. Cells were harvested 48 hrs post siRNA transfection. Individual siRNA sequences are listed in Supplementary Materials (Table S4).

### 4.3 Western Blotting

For analyzing siRNA efficiency by Western blot, cells grown in 12‐well plates were lysed in 120 µL 1X SDS loading buffer. After being boiled, the samples were separated on SDS‐PAGE gel and probed with the following antibodies: Rabbit anti‐TDP‐43 (Proteintech 10782‐2‐AP, 1:1000); Rabbit anti‐CELF2 (Proteintech 12921‐1‐AP, 1:1000); Mouse anti‐PTBP1 (Sigma Bb7, 1:6000); and Mouse anti‐GAPDH (Proteintech 60004‐1‐Ig, 1:4000).

### 4.4 Human Brain Samples and RNA Extraction

Postmortem frozen frontal cortex tissues from ALS patients and non‐neurological control subjects were obtained from the Johns Hopkins and Temple University ALS Postmortem Tissue Core. Subject demographics are provided in Table S5. The use of patient samples and data in this study was approved by Johns Hopkins University School of Medicine Office of Human Subjects Research Institutional Review Boards. For RNA extraction, tissues were homogenized in 1 mL of TRIzol reagent (Thermo Fisher Scientific, Cat. No. 15596018) using an Omni Tissue Master 125 homogenizer. Total RNA was then purified according to the standard Trizol method.

### 4.5 Semi‐Quantitative RT‐PCR

cDNA was synthesized using the HiScript III RT SuperMix Kit (Vazyme, Cat. No. R323‐01) with random hexamers, following the manufacturer's instructions. Semi‐quantitative PCR was performed using 2× LiTaq PCR Master Mix (LifeSct, Cat. No. M0024‐00) to assess splicing changes. For FOLH1 splicing analysis, the following primers were used: FOLH1‐splicing‐Forward 5’‐TGTGGTGGAGAAACTGGACC‐3’ and FOLH1‐splicing‐Reverse 5’‐TGTTCTCAGCTTTCAATTCATCCA‐3’. The PCR products were examined by 3% agarose gel electrophoresis and visualized using a ChemiDoc imaging system (Bio‐Rad).

### 4.6 RNA‐seq Data Alignment

The raw pair‐end reads were preprocessed to remove the adapters and perform quality control using the fastp program [58] (v.0.23.4). Reads were aligned to the human genome (hg38) with GENCODE v32 gene annotation using STAR aligner [59] (v.2.7.9a). Gene expression matrixes were computed by featureCounts [60] (v.2.0.1) command based on uniquely mapped reads.

### 4.7 Patients’ Bulk RNA‐seq Gene Expression and Splicing Analysis

Gene expression principal component analysis (PCA) was performed on TPM values of the top 10,000 genes with the largest standard deviation (SD). DEGs were calculated using DESeq2 [61], correcting for batch effects using the design: “∼ Batch + tissue + disease_group” and applying FDR correction.

Uniquely mapped bam files of each sample were used as input to MAJIQ [32] (v.2.4) for differential splicing analysis. Due to the strong heterogeneity among patients, it could not be treated as a simple repetition. We used the “heterogen” module to compare splicing junction usage between patients and control samples in specific tissues, and the output of the “heterogen” module was used as input to the “modulize” module to perform differential splicing events analysis, in which the threshold of ΔPSI was set to 0.1, and the threshold of *p*‐value was set to 0.05. To quantify the change of splicing junctions, we used custom R scripts to parse the output file of the “heterogen” module. The junctions with *p*‐values all less than 0.05, as calculated through the total number of mistakes (TNOM), t‐test, and Wilcoxon test, were identified as changed junctions, and among which, those with median PSI greater than the control group were considered up‐regulated, while those with median PSI less than the control group were considered down‐regulated. In postmortem patient samples, we defined the up‐regulated junctions with a median PSI less than 0.05 in control samples as aberrant junctions in specific tissue, and the other up‐regulated junctions were defined as normally used junctions.

Splicing PCA analysis was performed on PSI values of the top 10 000 junctions with the largest SD. Fisher's exact test was used to evaluate the statistical significance for the overlap of a Venn diagram on the 2 Í 2 contingency table, using the number of protein‐coding genes not in the diagram as the double negative set.

### 4.8 Bulk RNA‐seq Deconvolution Analysis

We used DWLS [22] to conduct deconvolution analysis on bulk RNA‐seq data from the frontal and temporal cortex of ALS, FTD, and PN samples, respectively. This analysis was based on the single‐cell level expression of known marker genes, which we obtained from The Human Protein Atlas. In our analysis, we included neuron marker genes such as the excitatory neuron marker gene SLC17A, as well as general neuron marker genes like MAP2 and TUBB3, among others. For oligodendrocytes, we included canonical marker genes such as OLIG and MBP. The detailed list of marker genes used for analysis was provided in Table S6.

### 4.9 Cell Line RNA‐seq Splicing Analysis

We used the “deltapsi” module to compare splicing junctions’ usage between knockdown and negative control samples and used custom R scripts to parse the output file to perform differential analysis of splicing junctions. The threshold of changed junctions was a *p*‐value less than 0.01 and an absolute value of ΔPSI more than 0.1. The novel junctions were defined through GENCODE annotation.

### 4.10 Patients’ snRNA‐seq Analysis

Single‐cell annotation metadata was downloaded from Synapse (SynID: syn51105515). All samples were merged into a Seurat object in the downstream analysis. The 60% of ALS patients’ motor cortex samples with the lowest oligodendrocyte content and c9FTD patients’ motor cortex samples were selected for differential gene expression analysis and cell‐cell communication analysis. We first normalized the count data of each cell using the “NormalizeData” function in the Seurat R package. Next, we applied the “FindMarkers” function to perform differential analysis on patients and PN samples. Genes exhibiting an absolute value of log2 fold change greater than 0.25 and *p*‐value less than 0.05 were identified as differentially expressed genes (DEGs).

The average expressions of neurons and glial cells were calculated in PN samples through the “AverageExpression” function in the Seurat R package. We used a strict threshold for identifying cell‐type‐specific expressed genes used for biomarker nomination, which required log2 fold change greater than 1, pct.1 greater than 0.7, and pct.2 less than 0.1.

Cell‐cell communications in motor cortex samples were inferred through the NeuronChat R package, and the method was described in the previous study [25].

### 4.11 Gene Enrichment Analysis of Splicing and Cell Type Expression

We calculated the odds ratio and *p*‐value for the overlap between ALS/FTD splicing‐altered genes and cell‐type‐specific expressed genes across different cell types within specific tissues using Fisher's exact test. The 2 × 2 contingency table was constructed based on the number of overlapping genes between ALS/FTD splicing‐altered genes and neuron‐ or glia‐specific expressed genes. Cell‐type‐specific expression was determined using the “FindMarkers” function on cells from PN samples, identifying genes with a log2 fold change > 1 and *p*‐value < 0.05 when compared to all other cell types. The enrichment heatmap was generated by calculating the proportion of overlapping genes relative to the total number of corresponding cell‐type‐specific expressed genes, and row‐wise normalization was applied to visualize the relative enrichment across cell types.

### 4.12 Disease Cell‐Type‐Specific Biomarkers Identification

In the frontal cortex, temporal cortex, motor cortex, and spinal cord of ALS‐TDP patients, all splicing junction biomarkers had been identified. FTD splicing junction biomarkers were detected in the frontal cortex and temporal cortex of FTD‐TDP patients. ALS‐associated splicing junction biomarkers must exhibit specific expression in oligodendrocytes and significant up‐regulation in ALS compared to control samples in at least one type of tissue. Conversely, FTD‐associated splicing junction biomarkers should specifically express in neurons and show significant up‐regulation in FTD compared to control samples in at least one type of tissue.

### 4.13 Random Forest (RF) Model Construction

The RF models were built using a combined set of ALS oligodendrocyte‐specific biomarkers and FTD neuron‐specific biomarkers. To ensure sample balance, we classified frontal cortex and temporal cortex samples from ALS‐TDP and FTD‐TDP patients based on the above ALS and FTD cell‐type‐specific biomarker features in RF models, respectively. Thirty percent of the samples were selected as test sets. Cell‐specific disease markers were chosen solely based on the training set data as model features. Besides, k‐fold cross‐validation was performed for feature selection and model training on each fold independently to test the model performance.

### 4.14 Abnormal Peptides Identification

We used MaxQuant [47] (v.2.4.9) software for mass spectrum (MS) search. The TMT MS raw data of ALS/FTD patients’ CSF were downloaded from Synapse (SynID: syn25795030, syn22059394). We predicted transcripts generated by aberrant splicing junctions in canonical transcripts annotated in the UCSC genome browser, removing annotated introns that had no intersection with the abnormal splicing junctions from the pre‐mRNA sequence. For each transcript sequence, the amino acid sequences of all possible open reading frames (ORFs) between every start codon and stop codon were extracted. Last, we merged predicted peptide sequences and the reviewed human protein sequences downloaded from Uniprot (https://www.uniprot.org) together to create the database for MS search. To obtain reliable results, we only defined protein groups that did not contain annotated proteins as abnormal peptides with translation evidence.

### 4.15 RNA Binding Protein (RBP) Binding Analysis

We downloaded human global RBP binding information through the POSTAR3 database [38]. If an RBP had binding sites in the 150 nt flanking region of the 5′ splice site and 3′ splice site of the junction, it was considered that this RBP bound the junction.

### 4.16 Statistical Analysis

Statistical analysis was performed in R Studio (R version 4.3.0), and graphs were plotted using the ggplot2 (v.3.4.3) R package. The number of samples and specific statistical tests performed were described in the figures or figure legends. The statistical significance of data was denoted on graphs by informing asterisks (*p‐value < 0.05, **p‐value < 0.01, ***p‐value < 0.001) or NS (not significant).

## Author Contributions

C.D., Y.L., and Y.Z. conceived the study. C.D., Y.S., and J.Y. performed the bioinformatics analysis. Y.L. performed the cell line experiments, and R.W. performed the validation experiments in human brain samples. C.D., Y.L., and Y.Z. wrote the manuscript. X.X. contributed important information. Y.Z. supervised the study.

## Conflicts of Interest

The authors declare no conflicts of interest.

## Supporting information




**Supporting File 1**: advs73895‐sup‐0001‐SuppMat.pdf.

**Supporting File 2**: advs73895‐sup‐0002‐TableS1.xlsx.

**Supporting File 3**: advs73895‐sup‐0003‐TableS7.xlsx.

**Supporting File 4**: advs73895‐sup‐0004‐TableS8.xlsx.

**Supporting File 5**: advs73895‐sup‐0005‐TableS9.xlsx.

**Supporting File 6**: advs73895‐sup‐0006‐TableS10.xlsx.

## Data Availability

The raw data files of patients’ bulk RNA‐seq are available in NCBI under accession code GSE153960, and the raw data files of patients’ snRNA‐seq are available in NCBI under accession code PRJNA1073234. RNA‐seq raw data files generated in this study are deposited in the Genome Sequence Archive (GSA) in BIG Data Center, Beijing Institute of Genomics (BIG), Chinese Academy of Sciences, under the accession code: HRA007269.

## References

[1] J. Phukan , M. Elamin , P. Bede , et al., “The Syndrome of Cognitive Impairment in Amyotrophic Lateral Sclerosis: A Population‐Based Study,” Journal of Neurology, Neurosurgery & Psychiatry 83, no. 1 (2012): 102–108, 10.1136/jnnp-2011-300188. 21836033

[2] J. Bang , S. Spina , and B. L. Miller , “Frontotemporal Dementia,” The Lancet 386, no. 10004 (2015): 1672–1682, 10.1016/S0140-6736(15)00461-4. PMC5970949 26595641

[3] J. R. Burrell , M. C. Kiernan , S. Vucic , and J. R. Hodges , “Motor Neuron Dysfunction in Frontotemporal Dementia,” Brain 134, no. Pt 9 (2011): 2582–2594, 10.1093/brain/awr195. 21840887

[4] C. Lomen‐Hoerth , T. Anderson , and B. Miller , “The Overlap of Amyotrophic Lateral Sclerosis and Frontotemporal Dementia,” Neurology 59, no. 7 (2002): 1077–1079, 10.1212/WNL.59.7.1077. 12370467

[5] P. Steinacker , P. Barschke , and M. Otto , “Biomarkers for Diseases With TDP‐43 Pathology,” Molecular and Cellular Neuroscience 97 (2019): 43–59, 10.1016/j.mcn.2018.10.003. 30399416

[6] A. L. Ji , X. Zhang , W. W. Chen , and W. J. Huang , “Genetics Insight Into the Amyotrophic Lateral Sclerosis/Frontotemporal Dementia Spectrum,” Journal of Medical Genetics 54, no. 3 (2017): 145–154, 10.1136/jmedgenet-2016-104271. 28087719

[7] H. Braak , J. Brettschneider , A. C. Ludolph , V. M. Lee , J. Q. Trojanowski , and K. Del Tredici , “Amyotrophic Lateral Sclerosis—A Model of Corticofugal Axonal Spread,” Nature Reviews Neurology 9, no. 12 (2013): 708–714, 10.1038/nrneurol.2013.221. 24217521 PMC3943211

[8] O. H. Tam , N. V. Rozhkov , R. Shaw , et al., “Postmortem Cortex Samples Identify Distinct Molecular Subtypes of ALS: Retrotransposon Activation, Oxidative Stress, and Activated Glia,” Cell Reports 29, no. 5 (2019): 1164–1177.e5, 10.1016/j.celrep.2019.09.066. 31665631 PMC6866666

[9] J. Eshima , S. A. O'Connor , E. Marschall , R. Bowser , C. L. Plaisier , and B. S. Smith , “Molecular Subtypes of ALS Are Associated With Differences in Patient Prognosis,” Nature Communications 14, no. 1 (2023): 95, 10.1038/s41467-022-35494-w. PMC9822908 36609402

[10] J. Li , M. K. Jaiswal , J.‐F. Chien , et al., “Divergent Single Cell Transcriptome and Epigenome Alterations in ALS and FTD Patients With C9orf72 Mutation,” Nature Communications 14, no. 1 (2023): 5714, 10.1038/s41467-023-41033-y. PMC10504300 37714849

[11] S. S. Pineda , H. Lee , M. J. Ulloa‐Navas , et al., “Single‐cell Dissection of the Human Motor and Prefrontal Cortices in ALS and FTLD,” Cell 187, no. 8 (2024): 1971–1989.e16, 10.1016/j.cell.2024.02.031. 38521060 PMC11086986

[12] L. M. Gittings , E. B. Alsop , J. Antone , et al., “Cryptic Exon Detection and Transcriptomic Changes Revealed in Single‐Nuclei RNA Sequencing of C9ORF72 Patients Spanning the ALS‐FTD Spectrum,” Acta Neuropathologica 146, no. 3 (2023): 433–450, 10.1007/s00401-023-02599-5. 37466726 PMC10412668

[13] P. M. McKeever , A. M. Sababi , R. Sharma , et al., “Single‐Nucleus Transcriptome Atlas of Orbitofrontal Cortex in ALS With a Deep Learning‐based Decoding of Alternative Polyadenylation Mechanisms,” Cell Genomics 5 (2025): 101007, 10.1016/j.xgen.2025.101007. 40967225 PMC12802741

[14] F. Limone , D. A. Mordes , A. Couto , et al., “Single‐nucleus Sequencing Reveals Enriched Expression of Genetic Risk Factors in Extratelencephalic Neurons Sensitive to Degeneration in ALS,” Nature Aging 4, no. 7 (2024): 984–997, 10.1038/s43587-024-00640-0. 38907103 PMC11257952

[15] H.‐L. V. Wang , J.‐F. Xiang , C. Yuan , et al., “pTDP‐43 Levels Correlate With Cell Type–Specific Molecular Alterations in the Prefrontal Cortex of C9orf72 ALS/FTD patients,” Proceedings of the National Academy of Sciences 122, no. 9 (2025): 2419818122, 10.1073/pnas.2419818122. PMC11892677 39999167

[16] S. Saez‐Atienzar , S. Bandres‐Ciga , R. G. Langston , et al., “Genetic Analysis of Amyotrophic Lateral Sclerosis Identifies Contributing Pathways and Cell Types,” Science Advances 7, no. 3 (2021): abd9036, 10.1126/sciadv.abd9036. PMC7810371 33523907

[17] J. Humphrey , S. Venkatesh , R. Hasan , et al., “Integrative Transcriptomic Analysis of the Amyotrophic Lateral Sclerosis Spinal Cord Implicates Glial Activation and Suggests New Risk Genes,” Nature Neuroscience 26, no. 1 (2023): 150–162, 10.1038/s41593-022-01205-3. 36482247

[18] K. E. Irwin , P. Jasin , K. E. Braunstein , et al., “A Fluid Biomarker Reveals Loss of TDP‐43 Splicing Repression in Presymptomatic ALS–FTD,” Nature Medicine 30, no. 2 (2024): 382–393, 10.1038/s41591-023-02788-5. PMC10878965 38278991

[19] S. Seddighi , Y. A. Qi , A.‐L. Brown , et al., “Mis‐Spliced Transcripts Generate De Novo Proteins in TDP‐43–Related ALS/FTD,” Science Translational Medicine 16, no. 734 (2024): adg7162, 10.1126/scitranslmed.adg7162. PMC11325748 38277467

[20] A. Sathyanesan , J. Zhou , J. Scafidi , D. H. Heck , R. V. Sillitoe , and V. Gallo , “Emerging Connections Between Cerebellar Development, Behaviour and Complex Brain Disorders,” Nature Reviews Neuroscience 20, no. 5 (2019): 298–313, 10.1038/s41583-019-0152-2. 30923348 PMC7236620

[21] P. Bede , R. H. Chipika , F. Christidi , et al., “Genotype‐associated Cerebellar Profiles in ALS: Focal Cerebellar Pathology and Cerebro‐cerebellar Connectivity Alterations,” Journal of Neurology, Neurosurgery & Psychiatry 92, no. 11 (2021): 1197–1205, 10.1136/jnnp-2021-326854. 34168085 PMC8522463

[22] D. Tsoucas , R. Dong , H. Chen , Q. Zhu , G. Guo , and G. C. Yuan , “Accurate Estimation of Cell‐Type Composition From Gene Expression Data,” Nature Communications 10, no. 1 (2019): 2975, 10.1038/s41467-019-10802-z. PMC6611906 31278265

[23] S. F. Sorrells , M. F. Paredes , A. Cebrian‐Silla , et al., “Human Hippocampal Neurogenesis Drops Sharply in Children to Undetectable Levels in Adults,” Nature 555, no. 7696 (2018): 377–381, 10.1038/nature25975. 29513649 PMC6179355

[24] B. Genç , J. H. Jara , S. S. Sanchez , et al., “Upper Motor Neurons are a Target for Gene Therapy and UCHL1 Is Necessary and Sufficient to Improve Cellular Integrity of Diseased Upper Motor Neurons,” Gene Therapy 29, no. 3–4 (2022): 178–192. 34853443 10.1038/s41434-021-00303-4 PMC9018479

[25] W. Zhao , K. G. Johnston , H. Ren , X. Xu , and Q. Nie , “Inferring Neuron‐neuron Communications From Single‐Cell Transcriptomics Through NeuronChat,” Nature Communications 14, no. 1 (2023): 1128, 10.1038/s41467-023-36800-w. PMC9974942 36854676

[26] A. M. Gomez , L. Traunmüller , and P. Scheiffele , “Neurexins: Molecular Codes for Shaping Neuronal Synapses,” Nature Reviews Neuroscience 22, no. 3 (2021): 137–151, 10.1038/s41583-020-00415-7. 33420412 PMC7612283

[27] T. C. Südhof , “Neuroligins and Neurexins Link Synaptic Function to Cognitive Disease,” Nature 455, no. 7215 (2008): 903–911. 18923512 10.1038/nature07456 PMC2673233

[28] S. Mora and I. Allodi , “Neural Circuit and Synaptic Dysfunctions in ALS‐FTD Pathology,” Frontiers in Neural Circuits 17 (2023): 1208876, 10.3389/fncir.2023.1208876. 37469832 PMC10352654

[29] K. A. Nave and H. B. Werner , “Myelination of the Nervous System: Mechanisms and Functions,” Annual Review of Cell and Developmental Biology 30, no. 1 (2014): 503–533, 10.1146/annurev-cellbio-100913-013101. 25288117

[30] S. C. Ling , M. Polymenidou , and D. W. Cleveland , “Converging Mechanisms in ALS and FTD: Disrupted RNA and Protein Homeostasis,” Neuron 79, no. 3 (2013): 416–438, 10.1016/j.neuron.2013.07.033. 23931993 PMC4411085

[31] J. P. Ling , O. Pletnikova , J. C. Troncoso , and P. C. Wong , “TDP‐43 Repression of Nonconserved Cryptic Exons Is Compromised in ALS‐FTD,” Science 349, no. 6248 (2015): 650–655, 10.1126/science.aab0983. 26250685 PMC4825810

[32] J. Vaquero‐Garcia , J. K. Aicher , S. Jewell , et al., “RNA Splicing Analysis Using Heterogeneous and Large RNA‐seq Datasets,” Nature Communications 14, no. 1 (2023): 1230, 10.1038/s41467-023-36585-y. PMC9984406 36869033

[33] K. Jaganathan , S. Kyriazopoulou Panagiotopoulou , J. F. McRae , et al., “Predicting Splicing From Primary Sequence With Deep Learning,” Cell 176, no. 3 (2019): 535–548.e24, 10.1016/j.cell.2018.12.015. 30661751

[34] Y. H. Jeong , J. P. Ling , S. Z. Lin , et al., “Tdp‐43 Cryptic Exons Are Highly Variable Between Cell Types,” Molecular Neurodegeneration 12, no. 1 (2017): 13, 10.1186/s13024-016-0144-x. 28153034 PMC5289002

[35] T. Kurosaki , M. W. Popp , and L. E. Maquat , “Quality and Quantity Control of Gene Expression by Nonsense‐Mediated mRNA Decay,” Nature Reviews Molecular Cell Biology 20, no. 7 (2019): 406–420, 10.1038/s41580-019-0126-2. 30992545 PMC6855384

[36] A.‐L. Brown , O. G. Wilkins , M. J. Keuss , et al., “TDP‐43 Loss and ALS‐risk SNPs Drive Mis‐Splicing and Depletion of UNC13A,” Nature 603, no. 7899 (2022): 131–137, 10.1038/s41586-022-04436-3. 35197628 PMC8891020

[37] X. R. Ma , M. Prudencio , Y. Koike , et al., “TDP‐43 Represses Cryptic Exon Inclusion in the FTD–ALS Gene UNC13A,” Nature 603, no. 7899 (2022): 124–130, 10.1038/s41586-022-04424-7. 35197626 PMC8891019

[38] W. Zhao , S. Zhang , Y. Zhu , et al., “POSTAR3: An Updated Platform for Exploring Post‐Transcriptional Regulation Coordinated by RNA‐binding Proteins,” Nucleic Acids Research 50, no. D1 (2022): D287–D294, 10.1093/nar/gkab702. 34403477 PMC8728292

[39] G. Meister , “Argonaute Proteins: Functional Insights and Emerging Roles,” Nature Reviews Genetics 14, no. 7 (2013): 447–459, 10.1038/nrg3462. 23732335

[40] S. Kervestin and A. Jacobson , “NMD: A Multifaceted Response to Premature Translational Termination,” Nature Reviews Molecular Cell Biology 13, no. 11 (2012): 700–712, 10.1038/nrm3454. 23072888 PMC3970730

[41] J. DeGeer and N. Lamarche‐Vane , “Rho GTPases in Neurodegeneration Diseases,” Experimental Cell Research 319, no. 15 (2013): 2384–2394, 10.1016/j.yexcr.2013.06.016. 23830879

[42] M. Prudencio , J. Humphrey , S. Pickles , et al., “Truncated Stathmin‐2 Is a Marker of TDP‐43 Pathology in Frontotemporal Dementia,” Journal of Clinical Investigation 130, no. 11 (2020): 6080–6092, 10.1172/JCI139741. 32790644 PMC7598060

[43] D. J. Bacich , K. M. Wozniak , X.‐C. M. Lu , et al., “Mice Lacking Glutamate Carboxypeptidase II Are Protected From Peripheral Neuropathy and Ischemic Brain Injury,” Journal of Neurochemistry 95, no. 2 (2005): 314–323, 10.1111/j.1471-4159.2005.03361.x. 16190866

[44] M. A. van Es , O. Hardiman , A. Chio , et al., “Amyotrophic Lateral Sclerosis,” The Lancet 390, no. 10107 (2017): 2084–2098, 10.1016/S0140-6736(17)31287-4. 28552366

[45] S. Li and Z. H. Sheng , “Oligodendrocyte‐derived Transcellular Signaling Regulates Axonal Energy Metabolism,” Current Opinion in Neurobiology 80 (2023): 102722, 10.1016/j.conb.2023.102722. 37028201 PMC10225329

[46] R. Kabiljo , A. Iacoangeli , A. Al‐Chalabi , and I. Rosenzweig , “Amyotrophic Lateral Sclerosis and Cerebellum,” Scientific Reports 12, no. 1 (2022): 12586, 10.1038/s41598-022-16772-5. 35869263 PMC9307771

[47] S. Tyanova , T. Temu , and J. Cox , “The MaxQuant Computational Platform for Mass Spectrometry‐Based Shotgun Proteomics,” Nature Protocols 11, no. 12 (2016): 2301–2319, 10.1038/nprot.2016.136. 27809316

[48] U. Šušnjar , N. Škrabar , A. L. Brown , et al., “Cell Environment Shapes TDP‐43 Function With Implications in Neuronal and Muscle Disease,” Communications Biology 5, no. 1 (2022): 314. 35383280 10.1038/s42003-022-03253-8 PMC8983780

[49] J. R. Tollervey , T. Curk , B. Rogelj , et al., “Characterizing the RNA Targets and Position‐dependent Splicing Regulation by TDP‐43,” Nature Neuroscience 14, no. 4 (2011): 452–458, 10.1038/nn.2778. 21358640 PMC3108889

[50] M. Polymenidou , C. Lagier‐Tourenne , K. R. Hutt , et al., “Long Pre‐mRNA Depletion and RNA Missplicing Contribute to Neuronal Vulnerability From Loss of TDP‐43,” Nature Neuroscience 14, no. 4 (2011): 459–468, 10.1038/nn.2779. 21358643 PMC3094729

[51] A. Donde , M. Sun , J. P. Ling , et al., “Splicing Repression Is a Major Function of TDP‐43 in Motor Neurons,” Acta Neuropathologica 138, no. 5 (2019): 813–826, 10.1007/s00401-019-02042-8. 31332509 PMC6802294

[52] H. Spence , F. M. Waldron , R. S. Saleeb , et al., “RNA Aptamer Reveals Nuclear TDP‐43 Pathology Is an Early Aggregation Event That Coincides With STMN‐2 Cryptic Splicing and Precedes Clinical Manifestation in ALS,” Acta Neuropathologica 147, no. 1 (2024): 50, 10.1007/s00401-024-02705-1. 38443601 PMC10914926

[53] A. Meneses , S. Koga , J. O'Leary , D. W. Dickson , G. Bu , and N. Zhao , “TDP‐43 Pathology in Alzheimer's Disease,” Molecular Neurodegeneration 16, no. 1 (2021): 84, 10.1186/s13024-021-00503-x. 34930382 PMC8691026

[54] S. H. Kang , Y. Li , M. Fukaya , et al., “Degeneration and Impaired Regeneration of Gray Matter Oligodendrocytes in Amyotrophic Lateral Sclerosis,” Nature Neuroscience 16, no. 5 (2013): 571–579, 10.1038/nn.3357. 23542689 PMC3637847

[55] A. Yousef , J. L. Robinson , D. J. Irwin , et al., “Neuron Loss and Degeneration in the Progression of TDP‐43 in Frontotemporal Lobar Degeneration,” Acta Neuropathologica Communications 5, no. 1 (2017): 68, 10.1186/s40478-017-0471-3. 28877758 PMC5586052

[56] M. Hruska‐Plochan , V. I. Wiersma , K. M. Betz , et al., “A Model of Human Neural Networks Reveals NPTX2 pathology in ALS and FTLD,” Nature 626, no. 8001 (2024): 1073–1083, 10.1038/s41586-024-07042-7. 38355792 PMC10901740

[57] C. Cividini , S. Basaia , E. G. Spinelli , et al., “Amyotrophic Lateral Sclerosis–Frontotemporal Dementia,” Neurology 98, no. 4 (2022): e402–e415, 10.1212/WNL.0000000000013123. 34853179 PMC8793105

[58] S. Chen , Y. Zhou , Y. Chen , and J. Gu , “fastp: An Ultra‐fast All‐in‐One FASTQ Preprocessor,” Bioinformatics 34, no. 17 (2018): i884–i890, 10.1093/bioinformatics/bty560. 30423086 PMC6129281

[59] A. Dobin , C. A. Davis , F. Schlesinger , et al., “STAR: Ultrafast Universal RNA‐seq Aligner,” Bioinformatics 29, no. 1 (2013): 15–21, 10.1093/bioinformatics/bts635. 23104886 PMC3530905

[60] Y. Liao , G. K. Smyth , and W. Shi , “featureCounts: An Efficient General Purpose Program for Assigning Sequence Reads to Genomic Features,” Bioinformatics 30, no. 7 (2014): 923–930, 10.1093/bioinformatics/btt656. 24227677

[61] M. I. Love , W. Huber , and S. Anders , “Moderated Estimation of Fold Change and Dispersion for RNA‐seq Data With DESeq2,” Genome Biology 15, no. 12 (2014): 550, 10.1186/s13059-014-0550-8. 25516281 PMC4302049

