# Supplementary material for: Aberrant Splicing Signatures Underpin Oligodendrocyte Damage in ALS and Neuron Loss in FTD
Source: Adv Sci (Weinh). 2026 Feb 4;13(17):e14886. doi: 10.1002/advs.202514886 (PMC13042764; doi:10.1002/advs.202514886)

Supplemental Information for

Aberrant Splicing Signatures Underpin Oligodendrocyte Damage in ALS and Neuron Loss in FTD

*Chen Du#, Yinming Li#, Rong Wu#, Yufei Shen, Jiayi Yang, Xuan Xiao, Yu Zhou**

The Supplemental Information file includes 9 supplementary figures and 10 supplementary tables.

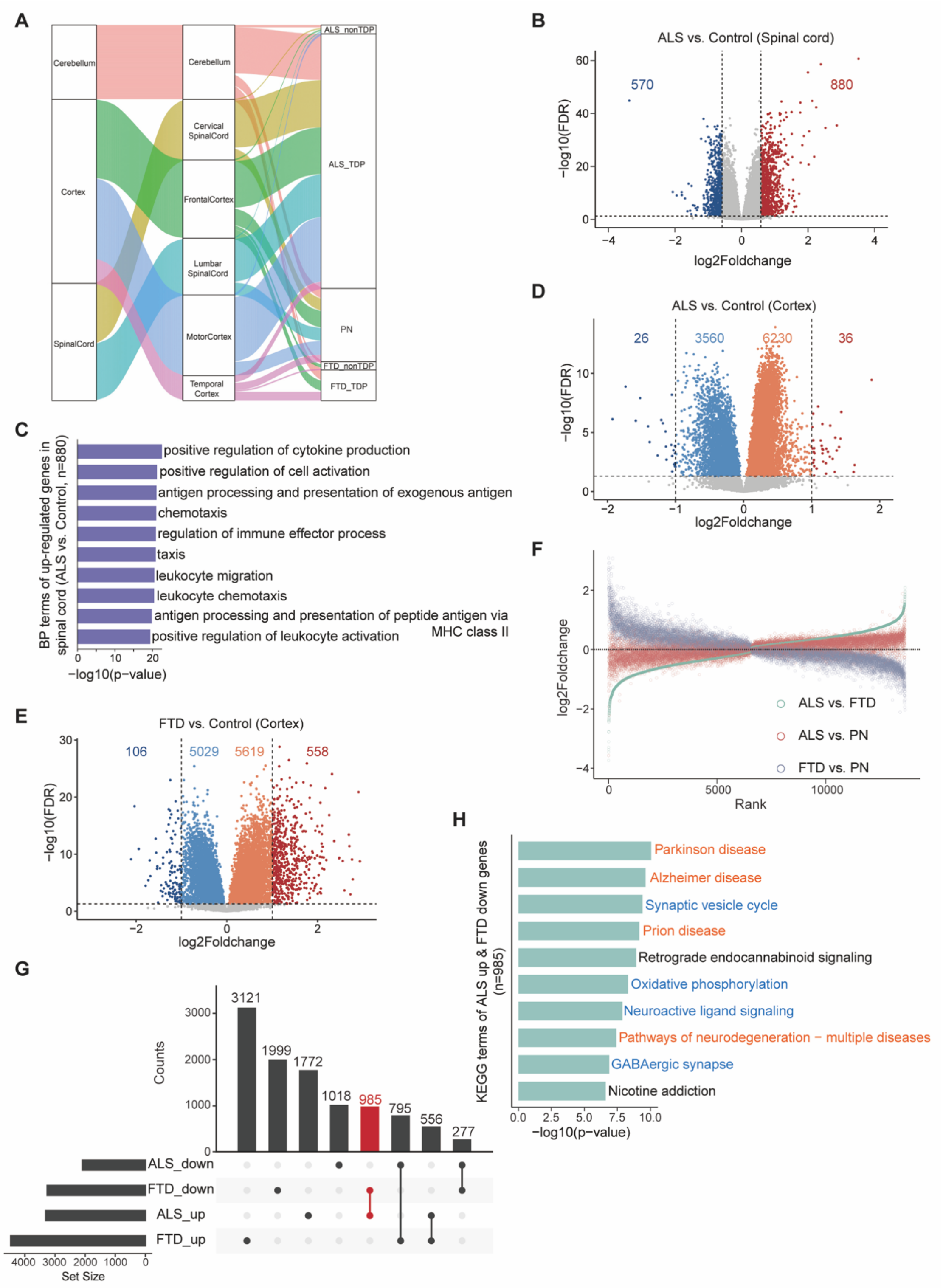

Supplementary Fig. S1. Bulk RNA-seq data overview and an expression atlas of ALS/FTD patients in multiple tissues, related to Fig. 1. (**A**) Sankey diagram of bulk RNA-seq samples distribution. (**B**) Volcano plot showing differential gene expression between ALS and normal control in the spinal cord. (**C**) Bar plot showing the top 10 GO-terms (BP) enriched in up-regulated genes identified in the spinal cord of ALS

patients. (**D-E**) Volcano plot showing differential gene expression between ALS and normal control (D), FTD and normal control (E) in the frontal and temporal cortex. (**F**) Scatter plot showing fold change of DEGs in the frontal and temporal cortex across three comparisons: ALS vs. FTD, ALS vs. PN, and FTD vs. PN. (**G**) Upset plot of DEGs of ALS and FTD in the frontal and temporal cortex. (**H**) Bar plot showing the top 10 KEGG-terms enriched in genes up-regulated in ALS but down-regulated in FTD in the frontal and temporal cortex.

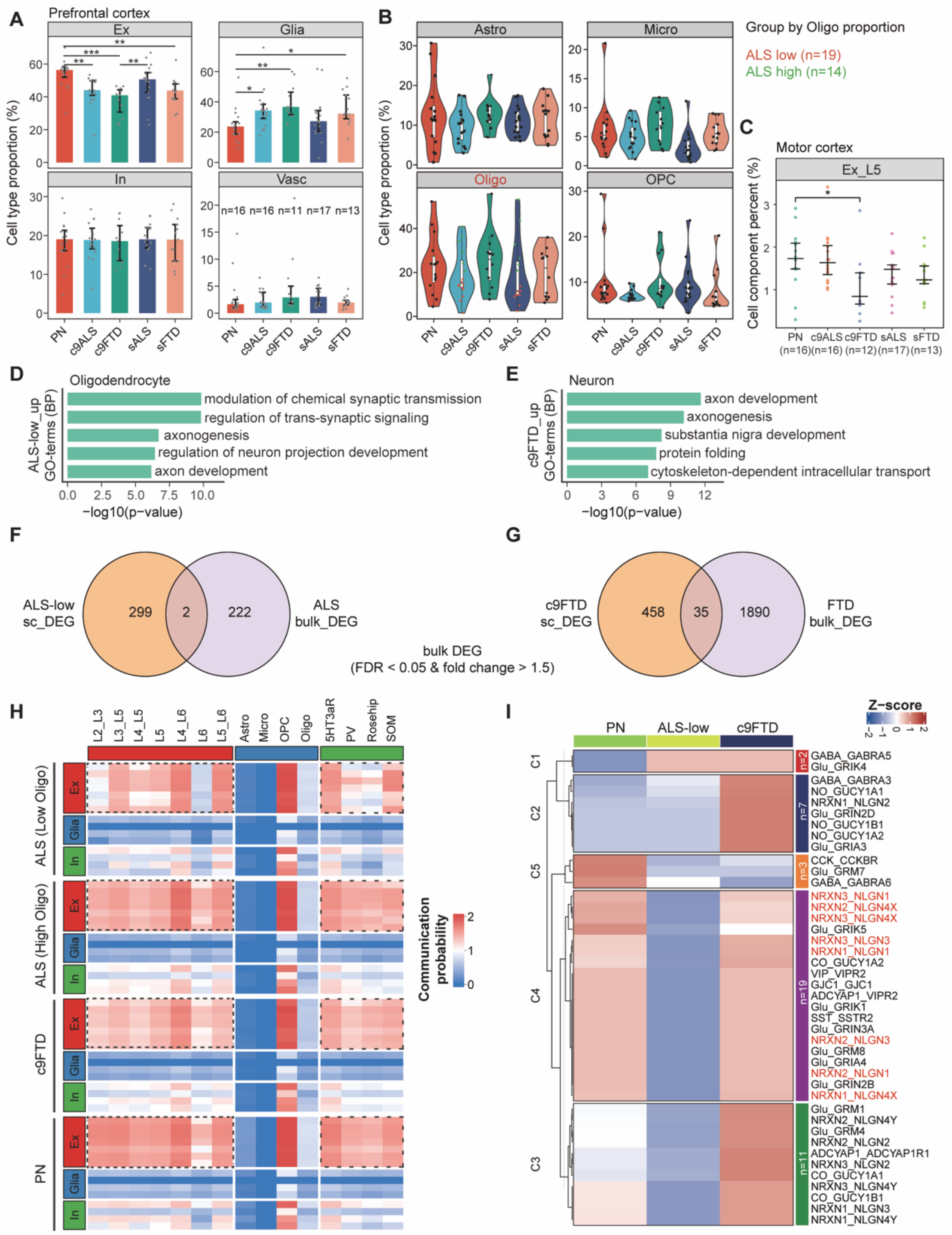

Supplementary Fig. S2. Cell proportion, gene expression, and cell-cell communication analysis of ALS/FTD cortex snRNA-seq, related to Fig. 2. (**A**) Bar plots showing the median proportion of 4 main cell groups in PN, ALS, and FTD patients' prefrontal cortex. Error bars representing the 25th and 75th percentiles. (**B**) Violin plots showing the distribution of glia cell proportions across all samples. Each sample's value is marked with an individual point. (**C**) Scatter plot showing the

proportion of L5 Ex in PN, ALS and FTD samples. (**D-E**) Bar plots showing the top 5 enriched BP terms of up-regulated genes in ALS-low patients' oligodendrocytes (D) and up-regulated genes in c9FTD patients' neurons (E), respectively. (**F-G**) Venn plots depicting the intersection of DEGs identified in snRNA-seq (snRNA-seq DEGs) and bulk RNA-seq (bulk RNA-seq DEGs) of ALS (F) and FTD (G). (**H**) Heatmap showing aggregated communication networks in neurons and glial cells of different disease types in the motor cortex. Dashed black boxes indicate interaction groups significantly decreased in ALS-low patients. (**I**) Heatmap comparing the number of links among ALS-low, c9FTD, and PN for each interaction pair. The P-value was calculated by the Wilcoxon test.

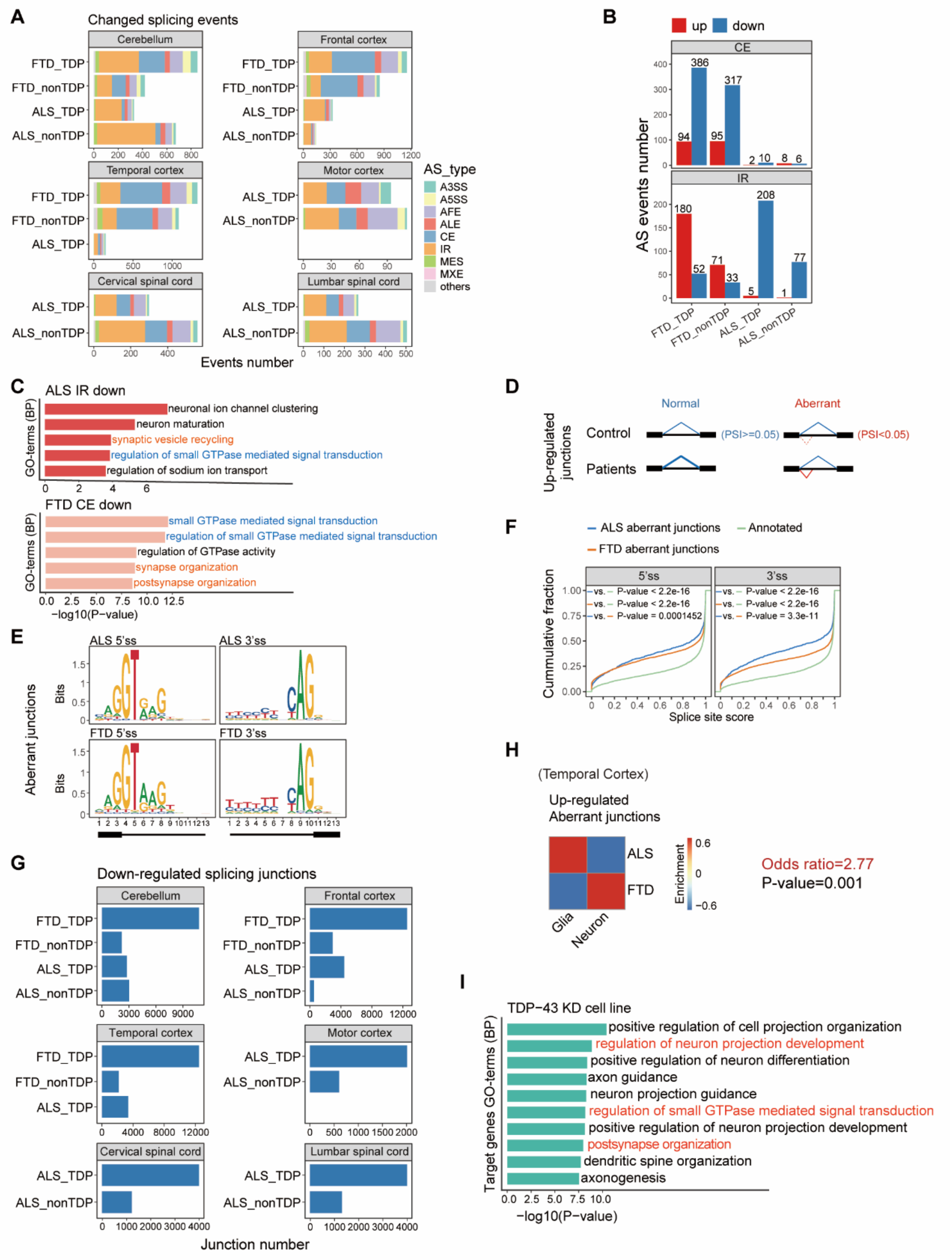

Supplementary Fig. S3. Changed splicing junctions and events detected in ALS and FTD samples, related to Fig. 3. (**A**) Bar plots showing the number of changed splicing events identified in different tissues between patients and PN. (**B**) Bar plots showing the number of changed CE and IR events identified in the frontal cortex between patients and PN. (**C**) Bar plots showing the top 5 enriched BP terms of genes harboring down-regulated IR in ALS (upper panel) and genes harboring down-

regulated CE in FTD (bottom panel). (**D**) Diagram of distinguishing aberrant and normal junctions in patients' up-regulated junctions. (**E**) Consensus sequences of ALS/FTD patients' aberrant splicing junctions aligned at 5′ ss (left) or 3′ ss (right). (**F**) Cumulative frequency distributions of SpliceAI-predicted 5′ ss scores (left) and 3′ ss scores (right) for ALS/FTD patients' aberrant junctions and annotated junctions. (**G**) Bar plots showing the number of down-regulated junctions identified in different tissues between patients and PN. (**H**) Heatmap showing the enrichment of ALS/FTD aberrant junctions in neurons and glial cells of the temporal cortex. Odds ratios and P-values are calculated by Fisher's exact test. (**I**) Bar plot showing the top 10 enriched BP terms of genes containing up-regulated novel junctions after TDP-43 knockdown in human cell lines.

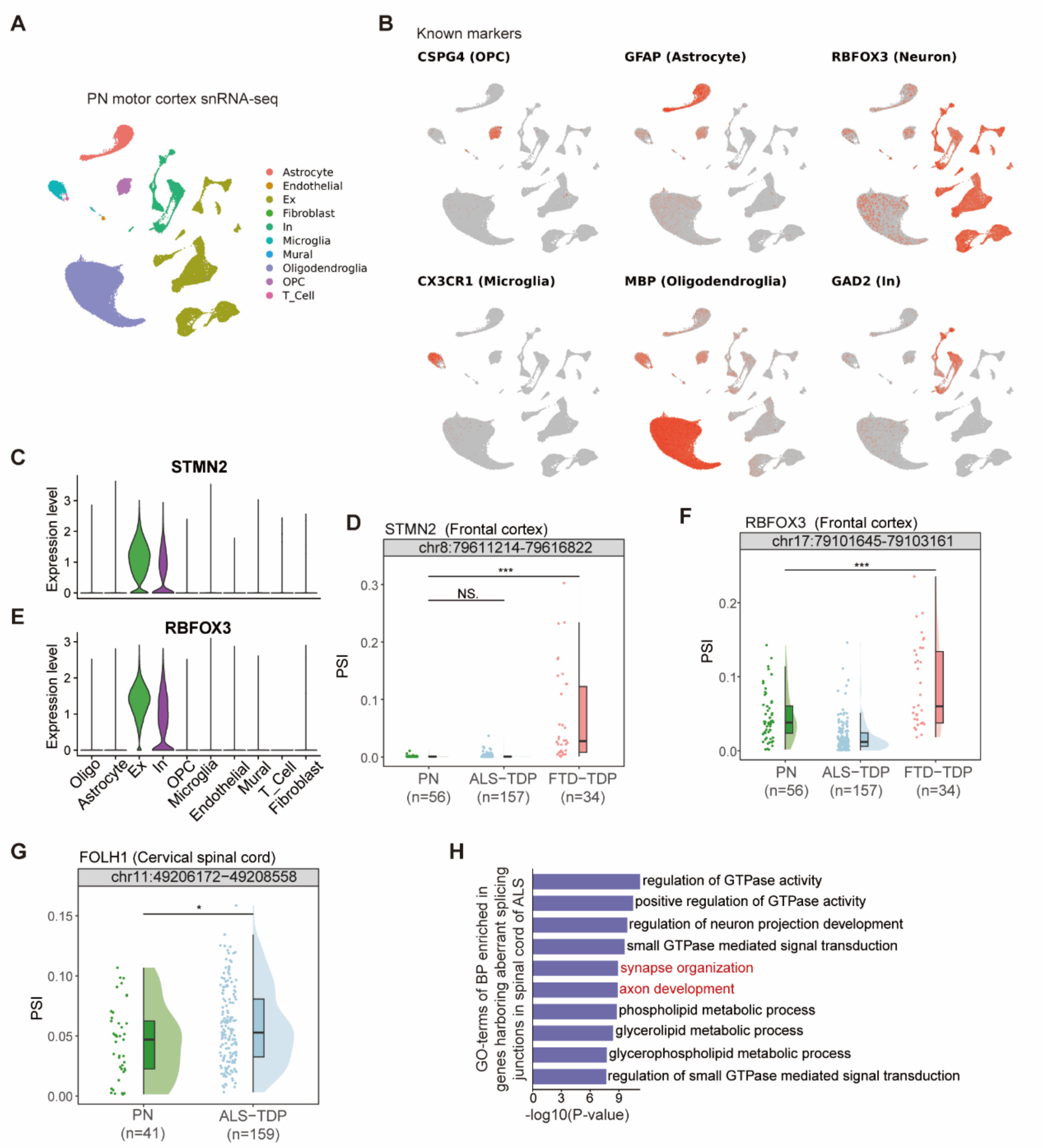

Supplementary Fig. S4. Quality control of snRNA-seq and cases of identified disease biomarkers in neuron or oligodendrocyte specific expression genes, related to Fig. 4. (**A**) UMAP plot of annotated cell clusters in PN motor cortex samples. (**B**) UMAP plots showing expression patterns of known canonical cell markers. (**C**) Single cell gene expression violin plots of STMN2. (**D**) Scatter and violin plots illustrating PSI changes of aberrant junctions in STMN2 among different groups in the frontal cortex. (**E-F**) Same as C-D for RBFOX3. (**G**) Scatter and violin plots illustrating PSI changes of the aberrant junction in FOLH1 between ALS and PN in the cervical spinal cord. (**H**) Bar plot showing the top 10 enriched BP GO-terms of genes harboring aberrant

junctions detected in ALS-TDP patients' spinal cords. The P-values were calculated by the Wilcoxon test.

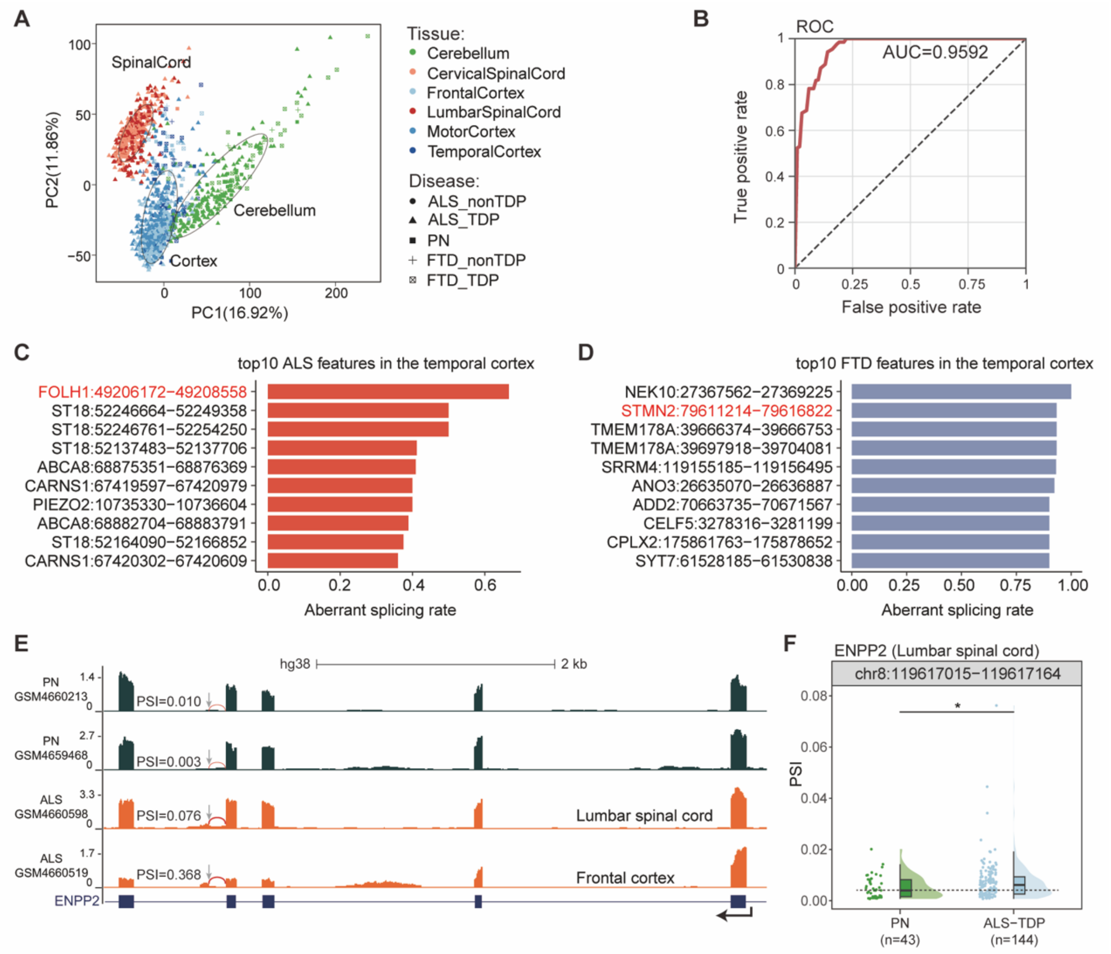

Supplementary Fig. S5. Cerebellum tissue-specific splicing pattern and abnormal peptides corresponding to splicing cases, related to Fig. 5. (**A**) PCA analysis of the splicing pattern of different patients and PN. The colors of dots represent tissues, and the shapes of dots indicate disease types. Black ovals indicated 60% confidence intervals of cerebellum, cortex, and spinal cord samples. (**B**) ROC curves of the RF model using disease-specific biomarkers as features through k-fold (5-fold) cross-validation. (**C-D**) Bar plots showing the top 10 ALS-specific (C) or FTD-specific (D) biomarkers with the highest rate in the temporal cortex samples of patients. (**E**) UCSC genome browser view of normalized patients' RNA-seq signals of ENPP2. Grey arrows indicated the novel splice site. The PSI values of the targeted junction are labeled for all samples, respectively. (**F**) Scatter and violin plots illustrating PSI changes of the aberrant junction in ENPP2 among different sample groups in the lumbar spinal cord. The P-values were calculated by the Wilcoxon test.

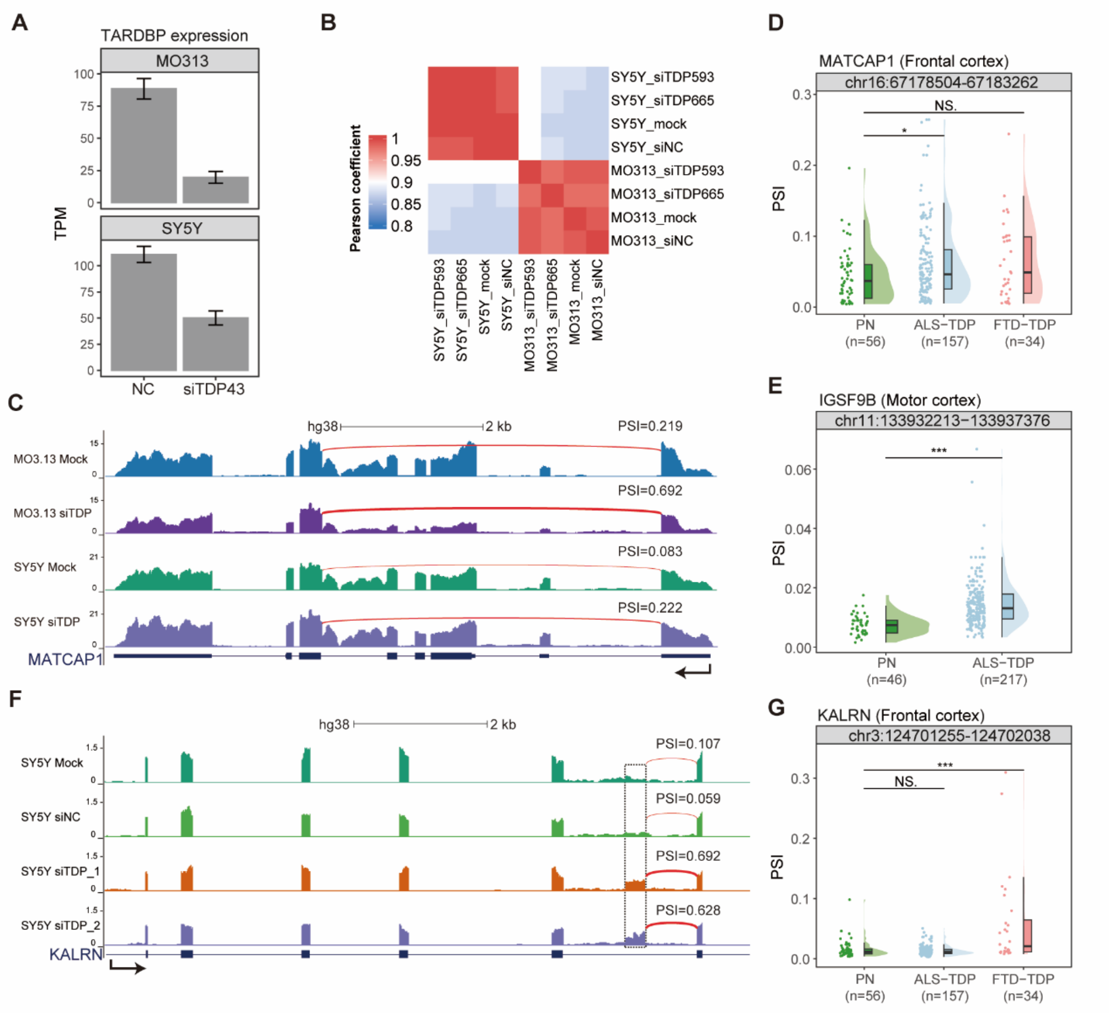

Supplementary Fig. S6. RNA-seq sample quality control and cases of identified disease biomarkers in specific splicing events of neuronal or oligodendrocyte cell lines affected by TDP-43 pathology, related to Fig. 7. (**A**) Bar plots showing TDP-43 knockdown effect at RNA level calculated through RNA-seq. (**B**) Pearson correlation among all samples based on gene expression. (**C**) UCSC genome browser view of normalized cell line RNA-seq signals of MATCAP1. The PSI values of the targeted junction are labeled for all samples, respectively. (**D**) Scatter and violin plots illustrating PSI changes of the aberrant junction in MATCAP1 among different patient groups in the frontal cortex. (**E**) Scatter and violin plots illustrating PSI changes of the aberrant junction in IGSF9B among different patient groups in the motor cortex. (**F**) UCSC genome browser view of normalized cell line RNA-seq signals of KALRN. The PSI values of the targeted junction are labeled for all samples, respectively. (**G**) Scatter and violin plots illustrating PSI changes of the aberrant junction in KALRN among

different patient groups in the frontal cortex. The P-values were calculated by the Wilcoxon test.

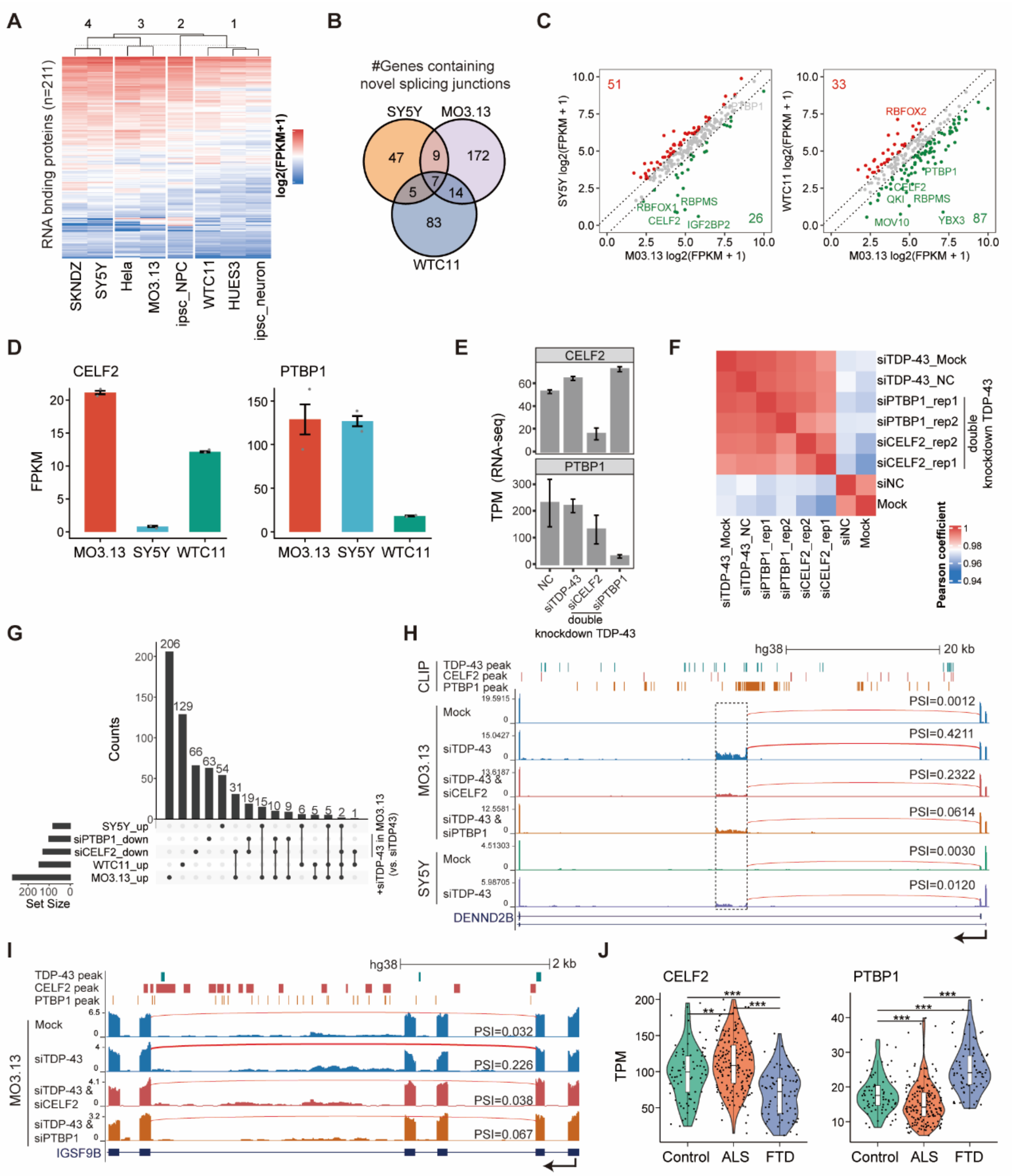

Supplementary Fig. S7. Data quality control and TDP-43 regulated splicing junctions promoted by PTBP1 or CELF2, related to Fig. 8. (**A**) Heatmap showing the characteristics of RBP expression in different cell lines. (**B**) Venn plot depicting the intersection of genes containing up-regulated novel splicing junctions after TDP-43 knockdown identified in SY5Y, WTC11, and MO3.13. (**C**) Scatter plots showing RBPs expression difference between MO3.13 and SY5Y (left panel), between MO3.13 and WTC11 (right panel). The dashed line indicated that the threshold for an obvious change with fold change greater than 1.5. (**D**) Bar plots showing the RNA expression level of CELF2 (left panel) and PTBP1 (right panel) among three cell lines. (**E**) Bar

plots showing PTBP1 and CELF2 knockdown effect at RNA level. (**F**) Pearson correlation among all samples based on gene expression (Mock was from cells transfected with the reagent only). (**G**) Upset plot showing the intersection of up-regulated novel splicing junctions after TDP-43 knockdown identified in SY5Y, WTC11, and MO3.13, down-regulated novel splicing junctions identified in PTBP1 or CELF2 knockdown under TDP-43 knockdown condition in MO3.13. (**H-I**) UCSC genome browser view of normalized RNA-seq signals of DENND2B (H) and IGSF9B (I). CLIP-seq peaks of TDP-43, CELF2, and PTBP1 from the POSTAR3 database are shown, and the PSI values of targeted junctions are labeled for all samples, respectively. (**J**) Violin plots showing the RNA expression level of CELF2 (left panel) and PTBP1 (right panel) among ALS, FTD and controls in the frontotemporal cortex.

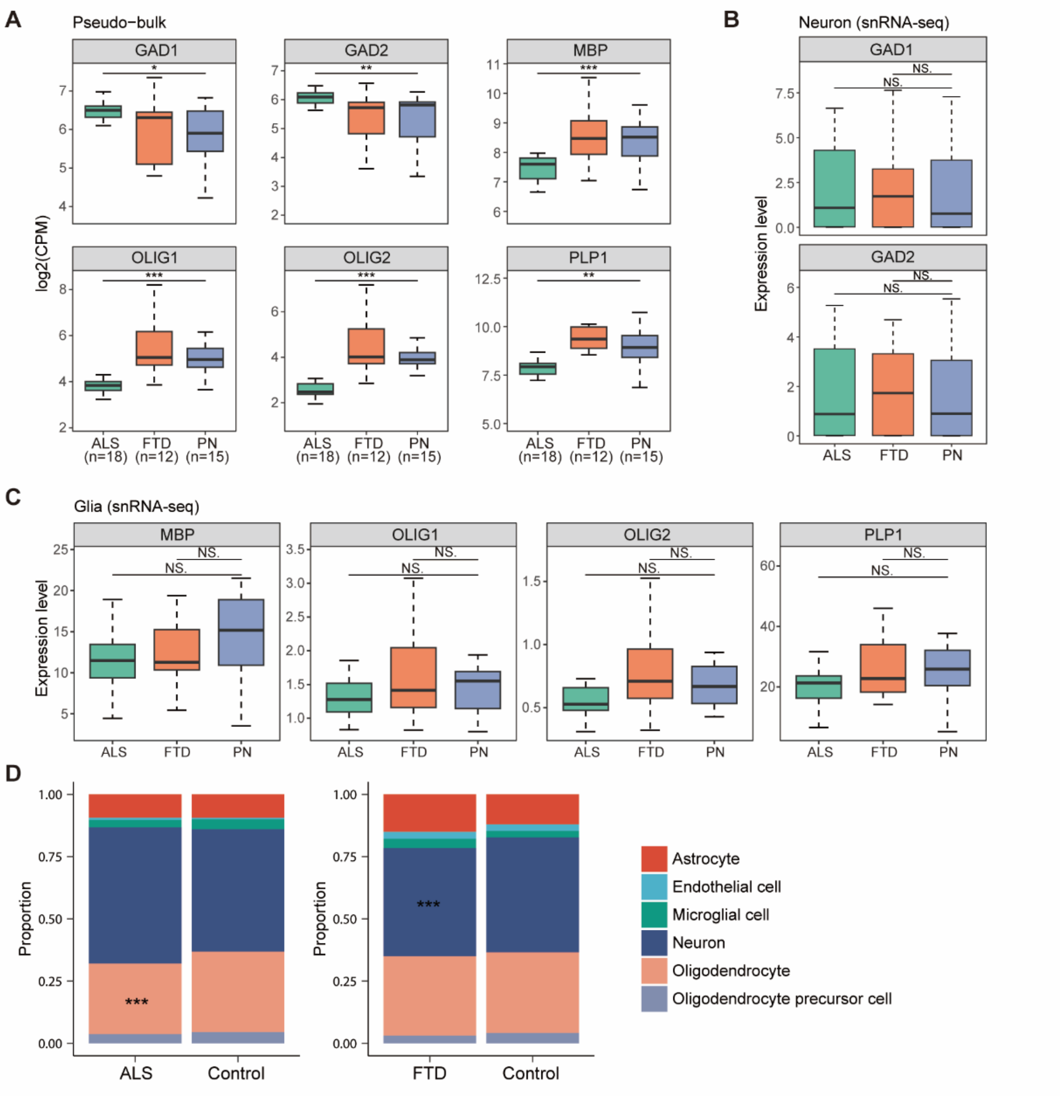

Supplementary Fig. S8. Canonical marker gene expression and cell-type composition in ALS/FTD patients' cortex from multiple dimensions. (**A**) Box plots showing the expression of canonical neuron marker genes (GAD1, GAD2), and canonical oligodendrocyte marker genes (MBP, OLIG1, OLIG2, PLP1) in ALS/FTD patients' motor cortex snRNA-seq merged pseudo-bulk RNA-seq. (**B**) Neuron marker genes' average single-cell expression of neurons between ALS, FTD, and PN. (**C**) Oligodendrocyte marker genes' average single-cell expression of glial cells between ALS, FTD, and PN. (**D**) Bar plots showing cell proportion across ALS and FTD in the frontal cortex. Original sequencing data are from Gittings et al., *Acta Neuropathol.*, 2023.

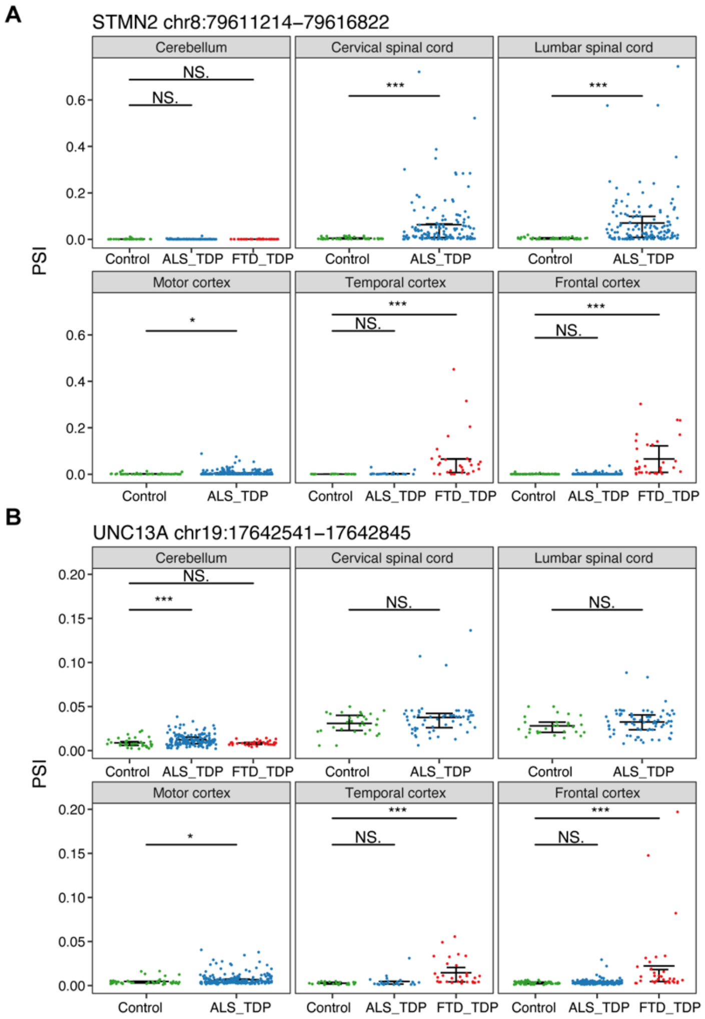

Supplementary Fig. S9. Aberrant splicing in STMN2 and UNC13A across distinct tissues. (**A-B**) Scatter plots showing PSI of STMN2 (A) and UNC13A (B) aberrant splicing across tissues.

Supplementary Tables

Supplementary Table S1. Fold change of DEGs with FDR < 0.05 in the cortex of ALS and FTD patients compared to controls (Excel file).

Supplementary Table S2. Information on public RNA-seq datasets of TDP-43 knockdown in human cell lines used in this study.

Cell line	Accession	URL	Origin
ipsc_neuron	GSE196144	https://www.ncbi.nlm.nih.gov/geo	iPSCs (201B7)
ipsc_NPC	GSE196144	https://www.ncbi.nlm.nih.gov/geo	iPSCs (201B7)
SKNDZ	ERP126666	https://www.ebi.ac.uk/ena/browser/home	
WTC11	ERP126666	https://www.ebi.ac.uk/ena/browser/home	
HUES3	GSE121569	https://www.ncbi.nlm.nih.gov/geo	
HeLa	GSE136366	https://www.ncbi.nlm.nih.gov/geo	

Supplementary Table S3. Genome coordinates of aberrant splicing junctions that generate abnormal peptides detected in CSF.

Chromosome	Start	End	junction_id	score	strand
chr11	106940178	106966357	GUCY1A2_2	.	-
chr1	183415276	183418183	NMNAT2_5	.	-
chr14	41742703	41766854	LRFN5_1	.	+
chr15	91281861	91289521	SV2B_4	.	+
chr17	79307824	79312364	RBFOX3_3	.	-
chr2	165091299	165130045	SCN3A_1	.	-
chr2	165092524	165094186	SCN3A_2	.	-
chr3	113603150	113603560	SIDT1_2	.	+
chr5	161366944	161410975	GABRB2_2	.	-
chr8	119617015	119617164	ENPP2_1	.	-
chr8	72919667	72935935	KCNB2_1	.	+
chr9	93268746	93278062	WNK2_2	.	+

chrX	110993482	111123077	PAK3_1	.	+

Supplementary Table S4. Sequences of siRNAs used in this study.

siRNA	Sequence
siPTBP1-1	CAGUUUACCUGuUUUUAAAtt
siPTBP1-2	GCAUCACGCUCUCGAAGCAtt
siCELF2-1	GCAAACCUUACUGAUCCUAtt
siCELF2-2	ACAAAUGGCAGGCAUGAAUtt
siTDP43-1	GCAAAGCCAAGAUGAGCCUtt
siTDP43-2	ACAAGUGAAAGUAAUGUCAtt

Supplementary Table S5. Demographic information for frozen postmortem tissues.

Patient GUID	Clinical Diagnosis	Age at time of collection	Gender
JHU100	Non-neurologic control	52	Female
JHU101	Non-neurologic control	70	Female
JHU123	Non-neurologic control	52	Male
JHU124	Non-neurologic control	85	Male
JHU129	Non-neurologic control	63	Female
JHU5	Sporadic ALS	67	Female
JHU6	Sporadic ALS	50	Female
JHU45	Sporadic ALS	56	Female
JHU87	Sporadic ALS	69	Female
JHU102	Sporadic ALS	72	Female
JHU107	Sporadic ALS	69	Male
JHU113	Sporadic ALS	67	Female
JHU16	C9ORF72-fALS	56	Female
JHU19	C9ORF72-fALS	52	Male
JHU22	C9ORF72-fALS	66	Male

JHU54	C9ORF72-fALS	54	Female
JHU92	C9ORF72-fALS	72	Male
JHU120	C9ORF72-fALS	68	Female
JHU86	C9ORF72-fALS/FTD	74	Male
JHU88	C9ORF72-fALS/FTD	59	Male
BRC2621	Sporadic FTD-TDP	64	Female
BRC2667	Sporadic FTD-TDP	74	Male
BRC2665	C9ORF72-FTD	62	Male
BRC2696	C9ORF72-FTD	64	Female

Supplementary Table S6. Marker genes for bulk RNA-seq deconvolution analysis.

Gene name	Cell type
CD31	Vascular cell
CD34	Vascular cell
CDH5	Vascular cell
VWF	Vascular cell
TEK	Vascular cell
ICAM1	Vascular cell
VCAM1	Vascular cell
LYVE1	Vascular cell
MAP2	Neuron
RBFOX3	Neuron
TUBB3	Neuron
NEFL	Neuron
SLC17A6	Neuron
SLC17A7	Neuron
GAD1	Neuron
GAD2	Neuron
OLIG1	Oligodendrocyte

OLIG2	Oligodendrocyte
MBP	Oligodendrocyte
GFAP	Astrocyte
VIM	Astrocyte
S100B	Astrocyte
IBA1	Microglia
TMEM119	Microglia
CX3CR1	Microglia

Supplementary Table S7. Genome coordinates of ALS oligodendrocyte-specific and FTD neuron-specific aberrant splicing junctions (Excel file).

Supplementary Table S8. Gene lists corresponding to the Venn diagrams in Figures 3G, 3L, 4I, and S7B.

Supplementary Table S9. Lists of neuron and glial cell marker genes.

Supplementary Table S10. List of abnormal peptides detected in the CSF.

Unprocessed blots

Figure 7A

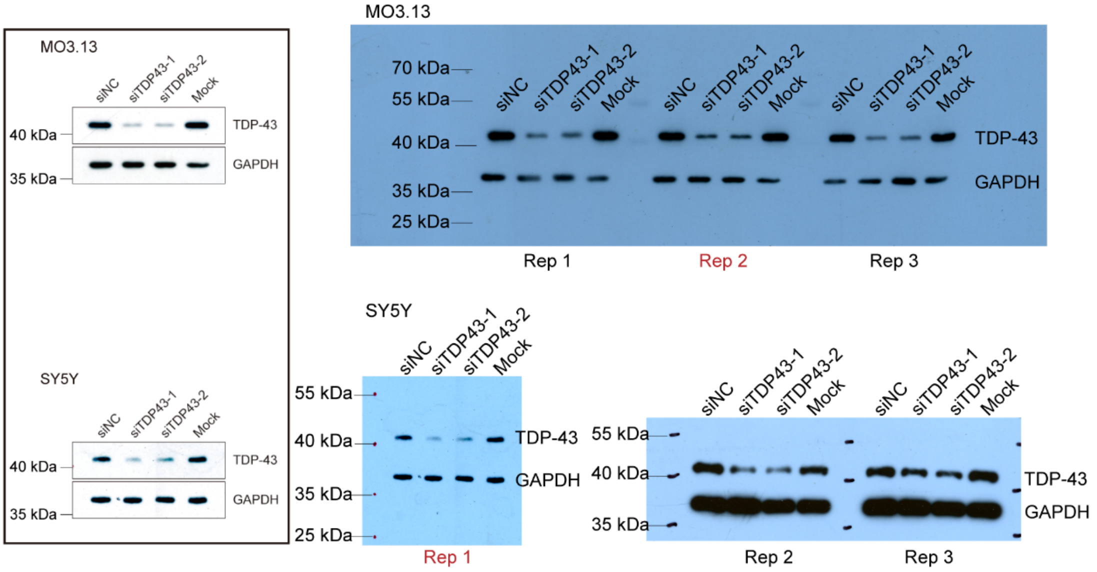

Figure 8E

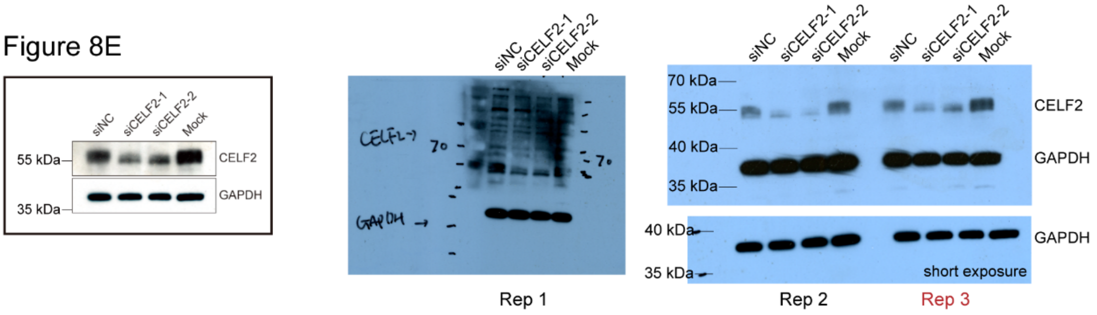

Figure 8F

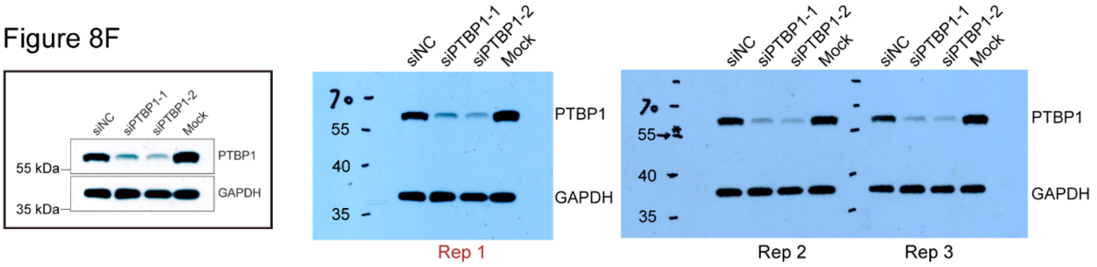

Supplement: Supplementary file 1 — Supporting File 1: advs73895‐sup‐0001‐SuppMat.pdf. [file ADVS-13-e14886-s005.pdf]
